# A scoping review of worldwide studies evaluating the effects of prehospital time on trauma outcomes

**DOI:** 10.1186/s12245-020-00324-7

**Published:** 2020-12-09

**Authors:** Alexander F. Bedard, Lina V. Mata, Chelsea Dymond, Fabio Moreira, Julia Dixon, Steven G. Schauer, Adit A. Ginde, Vikhyat Bebarta, Ernest E. Moore, Nee-Kofi Mould-Millman

**Affiliations:** 1 University of Colorado, Anschutz Medical Campus, 13001 E 17th Place, Aurora, CO 80045 USA; 2 United States Air Force Medical Corps, 7700 Arlington Boulevard, Falls Church, VA 22042 USA; 3 Denver Health and Hospital Authority, 777 Bannock St, Denver, CO 80204 USA; 4 Western Cape Government, Emergency Medical Services, 9 Wale Street, Cape Town, 8001 South Africa; 5 US Army Institute of Surgical Research, 3698 Chambers Rd., San Antonio, TX 78234 USA; 6 Ernest E. Moore Shock Trauma Center at Denver Health, 777 Bannock St, Denver, CO 80204 USA

**Keywords:** Prehospital time, Trauma, Emergency medical services

## Abstract

**Background:**

Annually, over 1 billion people sustain traumatic injuries, resulting in over 900,000 deaths in Africa and 6 million deaths globally. Timely response, intervention, and transportation in the prehospital setting reduce morbidity and mortality of trauma victims. Our objective was to describe the existing literature evaluating trauma morbidity and mortality outcomes as a function of prehospital care time to identify gaps in literature and inform future investigation.

**Main body:**

We performed a scoping review of published literature in MEDLINE. Results were limited to English language publications from 2009 to 2020. Included articles reported trauma outcomes and prehospital time. We excluded case reports, reviews, systematic reviews, meta-analyses, comments, editorials, letters, and conference proceedings. In total, 808 articles were identified for title and abstract review. Of those, 96 articles met all inclusion criteria and were fully reviewed. Higher quality studies used data derived from trauma registries. There was a paucity of literature from studies in low- and middle-income countries (LMIC), with only 3 (3%) of articles explicitly including African populations. Mortality was an outcome measure in 93% of articles, predominantly defined as “in-hospital mortality” as opposed to mortality within a specified time frame. Prehospital time was most commonly assessed as crude time from EMS dispatch to arrival at a tertiary trauma center. Few studies evaluated physiologic morbidity outcomes such as multi-organ failure.

**Conclusion:**

The existing literature disproportionately represents high-income settings and most commonly assessed in-hospital mortality as a function of crude prehospital time. Future studies should focus on how specific prehospital intervals impact morbidity outcomes (e.g., organ failure) and mortality at earlier time points (e.g., 3 or 7 days) to better reflect the effect of early prehospital resuscitation and transport. Trauma registries may be a tool to facilitate such research and may promote higher quality investigations in Africa and LMICs.

**Supplementary Information:**

The online version contains supplementary material available at 10.1186/s12245-020-00324-7.

## Introduction

Trauma is a time-sensitive condition which accounts for approximately 12% of the global burden of disease [1]. Trauma has significant health and economic implications that disproportionally affect populations in low- and middle-income countries (LMICs). Globally, over one billion people sustain traumatic injuries, and over six million die annually [1]. The injury mortality rate in LMICs (9–12%) is double the proportion seen in high-income countries (5.5%), and up to 16% of all disabilities globally are attributed to injury [1–6]. The median cost of direct medical expenditures related to injury in a study of LMICs was 15% of GDP per capita annually [7]. Despite advances in trauma care and expansion of prevention programs, injury and associated mortality rates continue to rise [1, 4, 8]. The US Military, for example, has policies and training based on research in prolonged field care; however, trauma care research focused on the resource-limited setting is necessary to reduce civilian trauma mortality and disability in these regions [5, 9–11].

Timely prehospital care is key to improving outcomes in time-sensitive injuries [12, 13]. The concept of timely prehospital trauma care and rapid transport has been a mainstay in prehospital teaching since Dr. R. Adams Cowley identified the preponderance of mortality within 1 h of traumatic injury [14]. There are relatively few published studies reporting patient outcomes directly due to prehospital care, and even fewer studies assessing the independent effects of prehospital time on patient mortality [15–18].

The relationship between prehospital time and patient outcomes remains unclear and conflicting [19, 20]. A 2014 systematic review focused on prehospital time and outcomes, performed by Harmsen et al., included 20 level III evidence articles and concluded a decrease in odds of mortality for the undifferentiated trauma patient when response time or transfer time are shorter, but conversely, there was an increased odds of survival with increased on-scene time and total prehospital time [18]. This conflict may be explainable by the heterogeneous nature of prehospital care and broad spectrum of disease pathophysiology in trauma. Additionally, most prehospital studies are conducted in high-income country (HIC) urban settings with limited generalizability to rural and LMIC environments. In rural and LMIC settings, where prehospital times can be very prolonged, understanding the impact, efficacy, timing, and effect size of specific prehospital interventions could lead to improved patient outcomes. Findings from additional research can help identify opportunities to improve systems and care, ultimately optimizing morbidity and mortality outcomes [13]. Many published trauma studies include aspects of prehospital care and time; however, this is typically not the primary focus of the study.

We seek to appraise the global scope of contemporary trauma literature focused on prehospital time and trauma patient outcomes in order to identify trends and gaps, which can directly inform recommendations on areas in need of further research.

## Methods

A scoping review of published literature was performed to critically appraise the relationship between trauma outcomes and prehospital time. A comprehensive literature search of MEDLINE, Embase, and Web of Science Core Collection databases was performed in January 2020. A combination of index terms and keywords including traumatic injury, prehospital time, and time to treatment were used to identify publications from 2009 to 2020 (Additional file 1: table 1). Results were limited to adult age group and exported to, and deduplicated in EndNote X9 (Clarivate Analytics, Philadelphia, PA). The Covidence systematic review software (Veritas Health Innovation, Melbourne, Australia) was used for screening and full text review.

For the first review, article abstracts were independently screened by two trained reviewers (AB, FM), blinded to each other’s reviews. Each reviewer read article titles and abstracts to determine if they satisfied inclusion criteria and to ensure they did not meet any exclusion criteria (see Table 1). Discrepant reviews of abstracts were adjudicated by a senior reviewer (NM).

**Table 1:** Screening and full-text article inclusion and exclusion criteria

Inclusion criteria	Exclusion criteria
Trauma-focused study or report	No hospital outcomes (morbidity or mortality outcomes)
Time (as a covariate, key exposure, or outcome)	Electrocution injuries
EMS-focused study*	Drowning injuries
Full text articles available	Focus on special populations (e.g., pediatrics, OB, incarcerated, psychiatric)
Adult patients	Field terminations (deceased on scene and not transported by EMS)
Published within the past 10 years	Case studies (or studies *N* < 50)
Articles written in English	Meta-analyses, systematic reviews, editorials, letters, and opinion pieces
	Abstract only, no full manuscript published

*Evidenced by EMS data, including vitals, transport modality, treatments, and/or transport time

Articles included after abstract review were divided between two reviewers (AB, LM) for a full text review and critical synthesis. The following key elements were assessed during each full text review: research questions, country, study design, injuries and populations studies, choice and definitions of independent and dependent variables, and level of evidence using GRADE criteria [21]. If any exclusion criteria were identified during full text review, the article was excluded with specific reason(s) provided (with approval from the senior reviewer). All included full text articles were coded into a summary table. Articles were grouped, based on common research categories, and one representative article from each category was summarized in a prose (paragraph) format. Articles not belonging to a specific category were individually summarized.

From the table of coded articles, key trends were descriptively reported using frequencies and percentages. Investigators independently appraised, then collectively discussed, all findings to reach consensus regarding key findings, conclusions, and recommendations which are presented qualitatively.

## Results

**Fig. 1:**
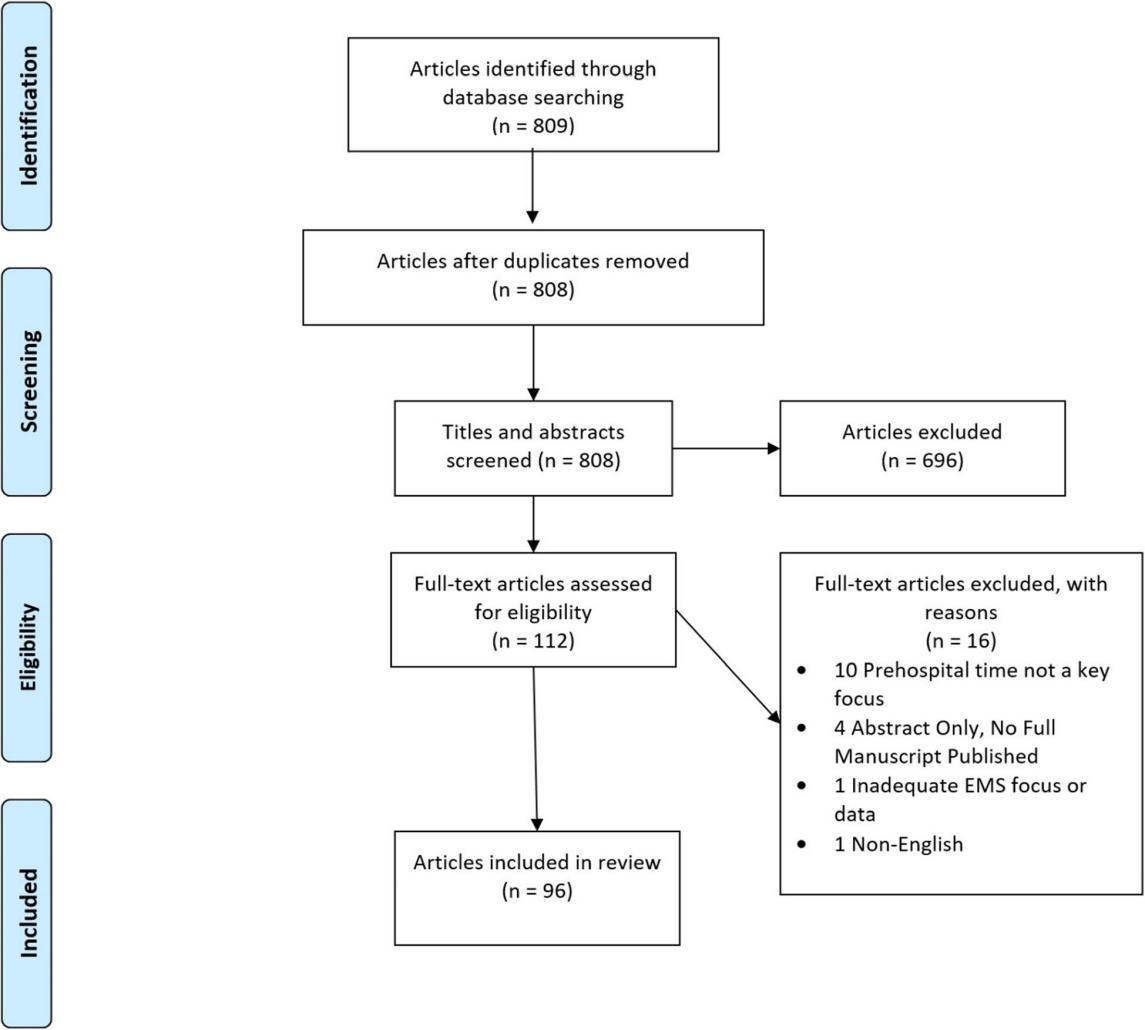
PRISMA [22] flowchart summarizing articles reviewed

We reviewed a total of 809 articles and included 96 after full text review (Fig. 1).

### Study characteristics

Of 96 articles included, the overwhelming majority (90, 94%) were observational with a few (6, 6%) being interventional in design (Table 2) [69, 78, 85, 88, 95, 98]. The six interventional studies evaluated the effects of prehospital blood product transfusion (plasma and packed red blood cells), and TXA administration on mortality, and used time (from injury to intervention) as a covariate. The largest proportion of articles originated from North America (42, 44%). Additional regions of origin included Europe (23, 24%), Asia (13, 14%), Australia (7, 7%), Africa (3, 3%), and South America (2, 2%). There were 6 (6%) articles of research simultaneously conducted in multiple geographic regions. We found 8 (8%) studies performed in LMICs, specifically Kenya, Malawi, Afghanistan, Iran, Iraq, and India. Of these, one study, conducted in Kenya, used a trauma registry as a data source [32]. The two studies in Afghanistan involve the US military patients only, as opposed to local trauma patients [72, 102]. The Iraqi studies, on the other hand, evaluated local prehospital trauma care and outcomes, aligning them more closely with other LMIC studies [86, 87].

**Table 2:** Coded summaries of included full text articles

Article reference	Category	EMS System	Setting	Country	Design	Time	Primary Outcome	Secondary Outcome	Primary Exposure(s)	Grade
Aiolfi (2018) [23]	Outcomes due to H-EMS vs G-EMS	Both	B	USA	O	Key exposure	In-hospital mortality	ICU LOS; hospital LOS	TBI; transport modality	Low
Al Thani (2014) [24]	Effect of PH intervention on outcomes	Both	B	Qatar	O	Covariate	PH and in-hospital mortality	-	Trauma; intubation	Low
Alarhayem et al. [25]	Miscellaneous	Both	B	USA	O	Key exposure	In-hospital mortality	-	Non-compressible torso trauma; PHT	Low
Anderson (2019) [26]	Miscellaneous	Both	C	Multiple	O	Key exposure	30-day survival	Predictors of survival	Traumatic cardiac arrest	Low
Andruszkow et al. [27]	Outcomes due to H-EMS vs G-EMS	Both	B	Germany	O	Covariate	In-hospital mortality	Multiple organ dysfunction syndrome and/or sepsis	Transport modality	Low
Bagher et al. [28]	G-EMS: time/distance vs mortality	G-EMS	CU	Sweden	O	Key exposure	Mortality	-	Total PH time, on scene time, PH rescue times	Low
Berlot et al. [29]	Outcomes due to H-EMS vs G-EMS	Both	B	Italy	O	Key exposure	Survival to discharge neurologic disability	-	TBI; transport modality	Low
Borst et al. [30]	Outcomes due to H-EMS vs G-EMS	Both	B	USA	O	Key exposure	In-hospital mortality	-	Trauma center transfer; transport modality	Low
Boschini (2016) [31]	Mortality due to primary vs secondary transfer	Both	B	Malawi	O	Covariate	In-hospital mortality	-	Primary versus secondary transfer to tertiary trauma center	Low
Botchey et al. [32]	Miscellaneous	Both	B	Kenya	O	Covariate	In-hospital mortality	-	Trauma	Low
Boudreau (2019) [33]	Effect of PH intervention on outcomes	Air	CU	USA	O	Covariate	In-hospital mortality	VTE development	Trauma; PH TXA administration in H-EMS	Low
Brazinova et al. [34]	Physiologic variables predicting outcomes in TBI	Both	B	Austria	O	Covariate	In-hospital mortality	Favorable neurologic outcomes	TBI; recommended early interventions	Low
Brorsson et al. [35]	Physiologic variables predicting outcomes in TBI	Both	B	Sweden	O	Key exposure	Mortality at 3 months post injury	Neurologic outcomes based on Glasgow Outcome Scale	Severe TBI (GCS ≤ 8)	Very low
Brown et al. [36]	G-EMS: time/distance vs mortality	G-EMS	B	Australia	O	Key exposure	30 day mortality	Hospital LOS for 30 day survivors	PH total time > 60 min; prolonged time intervals in either response; on-scene; transport; total	Low
Brown et al. [37]	Outcomes due to H-EMS vs G-EMS	Air	B	Australia	O	Key exposure	In-hospital mortality	-	H-EMS transport; time intervals	Low
Brown et al. [37]	G-EMS: Time/distance vs mortality	Both	B	USA	O	Key exposure	In-hospital mortality	-	Total PH time > 20 min	Low
Brown (2011) [38]	Outcomes due to H-EMS vs G-EMS	Both	B	USA	O	Covariate	Survival to hospital discharge	Hospital LOS; ICU admission; mechanical ventilation; emergent operations	Interfacility transfer of trauma patients HEMS and GEMS	Low
Brown et al. [39]	Outcomes due to H-EMS vs G-EMS	Both	B	USA	O	Covariate	Survival to hospital discharge	Hospital resource utilization; ICU admission; mechanical ventilation	HEMS vs GEMS transport for trauma patients	Low
Bulger et al. [40]	Outcomes due to H-EMS vs G-EMS	Both	B	USA	O	Key exposure	24 h survival	Survival to 28 days; 6-month GOS	Transport modality; hypovolemic shock; severe TBI	Low
Byrne et al. [41]	G-EMS: time/distance vs mortality	G-EMS	CU	USA	O	Key exposure	ED mortality	In-hospital mortality	PH time	Low
Cardoso (2014) [42]	Miscellaneous	Air	B	Brazil	O	Covariate	In-hospital mortality	Hospital length of stay	HEMS transport for trauma	Low
Chen (2014) [43]	Effect of PH intervention on outcomes	Both	CU	Taiwan	O	Covariate	Survival to hospital admission	Survival to hospital discharge	PH traumatic cardiac arrest with epinephrine administration	Low
Chen (2018) [44]	Outcomes due to H-EMS vs G-EMS	Both	B	USA	O	Key exposure	In-hospital survival	-	H-EMS vs. GEMS transport	Low
Chen et al. [45]	Time vs mortality	Both	B	USA	O	Key exposure	In-hospital mortality	-	PHT	Low
Chen et al. [45]	Miscellaneous	Both	B	Taiwan	O	Covariate	ROSC in the ED	30-day survival	Out of hospital traumatic cardiac arrest without PH ROSC	Low
Chiang et al. [46]	Effect of PH intervention on outcomes	Both	CU	Taiwan	O	Covariate	Survival to hospital admission	Survival to hospital discharge	PH traumatic cardiac arrest with epinephrine administration	Low
Chien (2016) [47]	Effect of PH intervention on outcomes	Both	B	Taiwan	O	Covariate	24-h survival	Survival to hospital discharge; cerebral function at discharge	Traumatic cardiac arrest receiving PH CPR	Low
Clark et al. [48]	Mortality due to rural vs urban	Both	B	USA	O	Covariate	In-hospital mortality	-	Trauma MVC	Low
Clements et al. [49]	Time vs mortality	Both	B	Canada	O	Key exposure	In-hospital mortality	Association between PHT and trauma team activation	All cause blunt trauma injury; EMS transport	Low
Crandall et al. [2]	Time vs mortality	Both	CU	USA	O	Outcome	In-hospital mortality	mean transport times	Gunshot victim > 5 miles from a trauma center	Low
deJongh (2012) [50]	H-EMS: time vs mortality	Air	B	Netherlands	O	Key exposure	In-hospital mortality	-	H-EMS vs. G-EMS transport; total PH time	Low
DeVloo (2018) [51]	Mortality due to primary vs secondary transfer	Both	CU	Belgium	O	Key exposure	30-day mortality	-	Primary vs secondary transfer to tertiary center; total time to tertiary center ED; skin incision for craniotomy	Low
Dinh et al. [15]	Time vs mortality	Both	B	Australia	O	Key exposure	In-hospital mortality	Survival to hospital discharge without requiring transfer for rehabilitation or nursing home care	Severe TBI (AIS ≥ 3); PH time	Low
Fatovich et al. [52]	Mortality due to rural vs urban	Both	B	Australia	O	Key exposure	In-hospital mortality	Hospital LOS	Major trauma; rural vs urban associated PH times	Low
Forristal (2018) [53]	Miscellaneous	Both	B	Canada	O	Covariate	Hypothermia (T < 35 °C) upon arrival to trauma center	Hospital LOS and survival to hospital discharge	EMS transport for severe trauma (ISS > 12)	Low
Foster et al. [54]	Outcomes due to H-EMS vs G-EMS	Both	B	USA	O	Covariate	Neurologic deterioration	ED disposition; in-hospital mortality; inter-facility transfer time; hospital LOS; nonroutine discharge; radiographic evidence of worsening spinal cord injury.	Spine injury with interfacility transfer; H-EMS vs G-EMS	Low
Franschman et al. [55]	Physiologic variables predicting outcomes in TBI	Both	B	Netherlands	O	Covariate	Neurologic deficit as determined by GOS	TBI-related mortality	TBI with transport to tertiary center; hypoxic or hypotensive events > 5 min during transport.	Low
Fuller et al. [56]	Time vs mortality	Both	B	UK	O	Key exposure	30-day inpatient mortality	-	EMS transport for severe TBI (AIS-head ≥ 3); EMS PHT intervals	Low
Fuller et al. [57]	Physiologic variables predicting outcomes in TBI	Both	B	UK	O	Key exposure	In-hospital mortality	Vital sign deterioration	TBI with transport to tertiary center; PHT intervals	Low
Funder et al. [58]	Time vs mortality	Both	CU	Denmark	O	Key exposure	30-day mortality	-	Penetrating trauma by EMS to trauma center; PHT	Low
Garcia (2017) [59]	Time vs mortality	G-EMS	CU	Canada	O	Key exposure	In-hospital mortality	-	Trauma with EMS transport to trauma center; PHT in intervals	Low
Gauss et al. [19]	Time vs mortality	Both	B	France	O	Key exposure	In-hospital mortality	-	Physician-staffed EMS to trauma center; PHT in intervals	Low
Gomes (2010) [60]	Effect of PH intervention on outcomes	Both	B	Portugal	O	Covariate	In-hospital mortality	-	Severe trauma requiring procedure; procedure done in PH; first hospital; arrival to trauma center	Low
Haltmeier et al. [61]	Effect of PH intervention on outcomes	Both	B	USA	O	Outcome	In-hospital mortality	Ventilator days; length of ICU stay; on-scene; PH time	Isolated severe blunt head injury (PH GCS ≤ 8) with or without PH intubation	Low
Hesselfeldt et al. [62]	H-EMS: mortality from physician vs paramedic	Air	B	Denmark	O	Outcome	Time from dispatch first ground EMS to arrival in the TC trauma bay	Proportion of severely injured patients secondarily transferred to the trauma center; 30-day mortality; on-scene triage.	Severe trauma patient transported by MD staffed H-EMS; PH fluid administration	Low
Hussmann et al. [63]	Effect of PH intervention on outcomes	Both	B	Germany	O	Covariate	In-hospital mortality	Sepsis; organ failure; multiple organ failure	Trauma with bleeding requiring transfusion > 1 unit pRBCs in hospital; PH fluid administration	Moderate
Hussmann et al. [64]	Effect of PH intervention on outcomes	Both	B	Germany	O	Covariate	In-hospital mortality	Hospital LOS; ICU LOS; ICU intubation; sepsis; organ failure; multi-organ failure	Level of PH fluid resuscitation of severe TBI patients	Low
Ingalls et al. [65]	H-EMS: time vs mortality	Air	C	Multiple	O	Key exposure	30-day mortality	Mortality en-route	Rapid evacuation by the Critical Care Air Transport (CCATT): time from wounding until time of arrival at the definitive care facility	Low
Jung et al. [66]	H-EMS: mortality from physician vs paramedic	Air	CU	South Korea	O	Covariate	Survival	TRISS	Group P patients transported by physician-staffed HEMS and group NP patients were transported by nonphysician-staffed HEMS	Low
Karrison (2018) [67]	G-EMS: time/distance vs mortality	G-EMS	CU	USA	O	Key exposure	ED/hospital mortality	None	Driving distance (shortest driving distance from the geocoded location of the scene of injury to the trauma center) transport time	Moderate
Kidher et al. [68]	H-EMS: time vs mortality	Air	CU	England	O	Key exposure	Mortality		Time-related variables, stay on scene time, arrival on scene time, total scene time	Moderate
Kim et al. [69]	Effect of PH intervention on outcomes	Air	CR	USA	I	Covariate	Mortality (overall and 24-h mortality)	Hospital stay; ICU LOS; ARDS, ARF	PH plasma administration	Moderate
Kim et al. [70]	G-EMS: time/distance vs mortality	G-EMS	Not specified	South Korea	O	Key exposure	In-hospital mortality		Scene time, PHT	Low
Klein (2019) [71]	Time vs mortality	Both	B	Multiple	O	Key exposure		Early SURG; ICU LOS; days intubated; organ failure; multiple organ failure; sepsis RISC prognosis; TRISS prognosis; in-hospital mortality; death within the first hour; death within the first 24 h; days of hospitalization	PH treatment time by intervals	Moderate
Kotwal et al. [72]	H-EMS: time vs mortality	Air	C	Afghanistan	O	Key exposure	Overall mortality, killed in action mortality, died of wound mortality	Amputation; cardiac arrest; coagulopathy; shock	Helicopter time < 60 min vs > 60 min	Moderate
Kotwal et al. [73]	Time vs mortality	Both	C	Multiple	O	Key exposure	Mortality		PH transport time, injury severity, blood transfusion	Moderate
Kulla et al. [74]	Miscellaneous	Both	B	Germany	O	Outcome	Trauma resuscitation time prolongation		Invasive emergency procedures	Low
Lansom et al. [75]	Effect of PH intervention on outcomes	Both	B	Australia	O	Outcome	Survival	Reduction in time from ED arrival to CT imaging	PH intubation compared with ED intubation	Low
Leis (2013) [76]	Effect of PH intervention on outcomes	G-EMS	CU	Spain	O	Key exposure	Survival to discharge		Response time	Low
Lovely et al. [77]	G-EMS: time/distance vs mortality	G-EMS	CR	USA	O	Key exposure	In-hospital mortality		PH scene time, PH transport time, Injury Severity Score (ISS)	Low
Lyon et al. [78]	Effect of PH intervention on outcomes	Air	B	England	I	Covariate	Mortality	ICU LOS	PRBC Transfusion	Low
Maddry et al. [79]	H-EMS: time vs mortality	Both	C	Not specified	O	Key exposure	Mortality up to 30 days	Morbidity up to 30 days, ICU and hospital stay	Time from the initial request for medical evacuation to arrival at a medical treatment facility	Moderate
Majidi et al. [80]	Physiologic variables predicting outcomes in TBI	Both	CU	USA	O	Covariate		Total hospital stay; in-hospital mortality; intensive care unit (ICU) days; ventilator days; discharge destinations	PH Neurologic Deterioration PHND	Moderate
Malekpour et al. [81]	Mortality due to primary vs secondary transfer	Both	CR	USA	O	Covariate	In-hospital mortality, ICU LOS, hospital LOS, complications	Pneumonia; pulmonary embolus; deep venous thrombosis; major arrhythmia, urinary tract infection, wound infection, acute renal failure	DA-direct admission IHT-Interhospital transfer	Moderate
McCoy (2013) [82]	G-EMS: time/distance vs mortality	G-EMS	CU	USA	O	Key exposure	In-hospital mortality		EMS on-scene and transport time intervals	Moderate
Meizoso et al. [83]	Effect of PH intervention on outcomes	Both	CU	USA	O	Outcome	Mortality on arrival (or DOA)		Intubation, needle decompression, tourniquet use, cricothyroidotomy, or advanced cardiac life support	Low
Middleton (2012) [84]	Miscellaneous	Both	B	Australia	O	Key exposure	Short-term neurological recovery (as determined by patient’s ASIA impairment scale grade on discharge from SCIU)	Deep vein thrombosis; pulmonary embolism; pressure ulcers	Time to definitive care center SCIU	Low
Möller et al. [20]	G-EMS: time/distance vs mortality	G-EMS	CU	South Africa	O	Key exposure	Mortality		Method of transport, hospital arrival time or PH transport time intervals	Low
Moore et al. [85]	Effect of PH intervention on outcomes	G-EMS	CU	USA	I	Outcome	Mortality	MOF at 28 days trauma-induced coagulopathy Shock Acute lung injury Exploratory outcomes: time from injury to need for first red blood cell transfusion Thromboelastography indices Number of ventilation free days Number of intensive-care-free days Development of MOF	Plasma administered in PH setting within 30 min of injury	High
Murad et al. [86]	G-EMS: time/distance vs mortality	G-EMS	B	Iraq	O	Key exposure	Mortality	Physiologic Severity Score	Assess 2 tier PH system (first responder and paramedic) vs no EMS in patients with long PHTs	Low
Murad et al. [87]	G-EMS: time/distance vs mortality	G-EMS	B	Iraq	O	Key exposure	Mortality		PH period intervals	Low
Neeki, et al. [88]	Effect of PH intervention on outcomes	Both	B	USA	I	Outcome	Mortality 24 h, 48 h, and 28 days	Total blood products transfused Hospital and ICU LOS, SBP prior to TXA administration, GCS prior to the first TXA dose in the field Adverse events	Prehospital TXA administration vs no TXA administration in patients with signs of h. shock	High
Newberry (2019) [89]	Miscellaneous	G-EMS	CR	India	O	Covariate	Mortality at 2, 7, and 30 days	Oxygen delivery; Intravenous fluids; functional status	Transport by EMS if burn injury	Low
Newgard et al. [90]	Outcomes due to H-EMS vs G-EMS	Both	B	Multiple	O	Key exposure	28-day mortality in shock, 6-month neurologic function in TBI		Total out-of-hospital time (time of initial 9-1-1 call to time of EMS arrival at the receiving hospital ED)	Moderate
Newgard (2010) [91]	Outcomes due to H-EMS vs G-EMS	Both	B	Multiple	O	Key exposure	Mortality		EMS time intervals	Moderate
Pakkanen et al. [92]	G-EMS: mortality from physician vs paramedic	Both	B	Finland	O	Covariate	Mortality, neurological outcome of TBI patients		EMS physician-staffed, EMS paramedic-staffed	Low
Paravar (2014) [93]	G-EMS: time/distance vs mortality	G-EMS	B	Iran	O	Key exposure	Mortality (in-hospital)		PHT advanced trauma life support interventions	Low
Prabhakaran et al. [94]	Mortality due to primary vs secondary transfer	Both	CU	USA	O	Outcome	Mortality in TBI	Time to arrival at a level I trauma center; time to initiation of multimodality neurophysiological monitoring; goal-directed therapy protocol	Scene to hospital vs transfer to hospital	Low
Pusateri et al. [95]	Effect of PH intervention on outcomes	Both	B	USA	I	Covariate	28-day mortality	24-h mortality; volumes of in-hospital blood components administered; ventilator-free days	PH transport times COMBAT Study pt. received plasma vs standard care PAMPer Study pt. received plasma vs standard care	Moderate
Raatiniemi (2015) [96]	Mortality due to rural vs urban	Air	B	Finland	O	Covariate	30-day mortality rate	Length of intensive care unit stay	Rural vs urban HEMS	Low
Rappold et al. [97]	Miscellaneous	G-EMS	CU	USA	O	Covariate	Mortality in hospital		ALS-transported trauma victims relative to BLS-transported trauma victims and among police-transported trauma victims	Low
Reitz et al. [98]	Effect of PH intervention on outcomes	Both	B	USA	I	Outcome	28-day mortality	24-h mortality; PH transport time; presenting indices of shock and coagulopathy units of in-hospital blood components administered	COMBAT study pt. received plasma vs standard care PAMPer Study pt. received plasma vs standard care	Moderate
Ruelas (2018) [99]	Time vs mortality	Both	B	USA	O	Key exposure	PH and ED mortality		PHT and procedures on penetrating trauma	Low
Ryb (2013) [100]	Outcomes due to H-EMS vs G-EMS	Both	B	USA	O	Covariate	Mortality		HEMS VS GEMS	Low
Seamon et al. [101]	Time vs mortality		CU	USA	O	Key exposure	Mortality		PHT prolonged by ALS vs BLS	Low
Shackelford et al. [102]	Effect of PH intervention on outcomes	Air	C	Afghanistan	O	Key exposure	Mortality at 24 h and 30 days	Prevalence of shock	Initiation of PH transfusion RBC, plasma, or both	Moderate
Spaite et al. [103]	Physiologic variables predicting outcomes in TBI	Both	CU	USA	O	Key exposure	Mortality in-hospital		Hypotension depth-duration out of hospital	Moderate
Talving (2009) [104]	Outcomes due to H-EMS vs G-EMS	Both	CU	USA	O	Covariate	Mortality	LOS; discharge time; ICU admission	HEMS vs. ground emergency medical service (GEMS) > 30 min	Low
Tansley (2019) [105]	G-EMS: time/distance vs mortality	G-EMS	B	Canada	O	Key exposure	Mortality		PH transfer time to trauma center	Low
Taylor (2018) [106]	Outcomes due to H-EMS vs G-EMS	Both	B	USA	O	Covariate	Mortality		HEMS vs. ground emergency medical service (GEMS)	Low
Tien (2011) [107]	G-EMS: time/distance vs mortality	G-EMS	CU	Canada	O	Key exposure	Hospital survival		PHT Time-to-surgery	Low
Weichenthal (2015) [108]	Effect of PH intervention on outcomes	Both	B	USA	O	Covariate	Survival to hospital discharge		Needle thoracostomy VS No Needle Thoracostomy	Low
Yeguiayan et al. [109]	G-EMS: mortality from physician vs paramedic	G-EMS	CU	France	O	Covariate	30-day mortality	72-h mortality	Physician EMS vs non-Physician EMS	Low
Zalstein (2010) [110]	Miscellaneous	Both	B	Australia	O	Covariate	Mortality	Adverse events	Patient inter-hospital transfer	Low
Zhu (2019) [111]	Miscellaneous	Both	B	USA	O	Covariate	Survival, LOS, ICU days, ventilator days		Pt that required mass transfusion protocol	Low
Zhu (2018) [112]	Outcomes due to H-EMS vs G-EMS	Both	CR	USA	O	Covariate	Survival to discharge from hospital		HEMS v GEMS	Low

### Trauma mechanism and bodily injuries

Most studies included any trauma mechanism, commonly defined as external force to the body not including bites, stings, burns, or drownings. A specific mechanism of injury was stated in the inclusion criteria in relatively few studies, and mechanism was often either “blunt” [49, 66, 98, 109] or “penetrating” [58, 97, 101], though some did look at motor vehicle collisions as a specific mechanism [48, 77]. There were several studies that focused on isolated torso injuries [25, 79], but overall, the majority of articles (73, 76%) included any trauma mechanism to any body part. The notable exceptions were 17 (18%) studies of head-injured patients, which assessed the effect of prehospital interventions and/or prehospital time on neurologic outcomes [29, 34, 35, 55, 57, 61, 75, 80, 90, 92, 94, 103].

### Main outcomes

Mortality was a primary outcome in the majority (90, 94%) of articles. Other frequently used primary outcomes included neurologic decline among head-injured patients [29, 54, 55, 90, 92], duration of trauma resuscitation [74], and EMS response times [62]. For most studies, in-hospital mortality was the most frequently used mortality outcome measure and was most often defined as all-cause death during hospital admission. Several articles assessed mortality within a specified period of time, starting as early as prehospital or ED mortality, and as far out as 3-months post-injury [35], although follow-up periods beyond 3 months were less commonly used. In traumatic brain injury (TBI) and spinal cord injury studies, neurologically focused outcomes were often the primary outcome while mortality was a secondary outcome [35, 54]. In neurologic trauma studies, survivors’ outcomes were assessed at discharge or long after admission (often 3 to 6 months) using neurologic functional outcome measures (e.g., Glasgow Outcome Scale score).

### Secondary outcomes

Secondary outcomes varied widely across articles, with the five most frequently used being hospital length of stay, intensive care unit (ICU) length of stay, days on mechanical ventilation, neurologic outcomes (most frequently Glasgow Outcome Scale), and EMS transport times (Table 2). Injury severity scoring measures were used in 73 (76%) articles to risk stratify and cohort similarly injured sub-groups of trauma patients, of which 54 (74%) used anatomic severity measures (injury severity score [ISS], abbreviated injury score [AIS], new injury severity score [NISS]); 3 (3%) used physiologic or hybrid scores (e.g., trauma injury severity score [TRISS]); and 17 (18%) used a combination of anatomic, physiologic, and/or hybrid scores (e.g., revised trauma score [RTS]). There were only a few studies that measured organ failure as a secondary outcome—four (4%) articles used multiple organ failure as a secondary outcome [27, 63, 64, 85] assessed by the Sequential Organ Failure Assessment (SOFA) score, and two (2%) studies specified acute renal failure as the organ failure outcome [69, 81].

### Prehospital time as a key exposure

Prehospital time, the primary variable of interest of this scoping review, was used as a key exposure (independent variable) in 48 (50%) articles. Prehospital time was most commonly defined as crude time from EMS notification to hospital arrival time. A common objective of these studies was to assess the effect of prehospital time (total time, or seldom, time intervals) on pre- or in-hospital mortality. Studies reported mixed (negative, neutral, and positive) associations with mortality with shorter prehospital times. Fatovich et al., in their study of urban and rural trauma patients in Western Australia, found that the risk of death was two times higher among the rural population when compared to urban trauma patients (rural population experienced significantly longer times to definitive care with median times of 11.6 h versus 59 min, respectively). They also identified no difference in mortality outcomes when the rural trauma patient survived to admission to a tertiary trauma center, when compared to the urban trauma patient [52]. Bagher et al. found that on-scene time (median 17 min, IQR 11–23 min) and total prehospital time (median 35 min, IQR 27–46 min) had no associated effect on mortality among urban prehospital transports in Scandinavia [28]. Similarly, Brown et al. found no association between prehospital time “of one hour and 30-day mortality” (adjusted OR 1.1, 95% CI 0.71–1.69), but did find association between scene times and longer hospital lengths of stay, with each additional minute of on-scene time associated with 1.16 times longer length of hospital stay (95% CI 1.03–1.31) [36]. Finally, when total prehospital time was sub-divided into intervals (response time, scene time, and transport time), Brown et al. found that there was an association (OR 1.21; 95% CI 1.02–1.44, *p* = 0.03) between prolonged scene time and mortality, regardless of transport modality (air or ground) [37]. Therefore, the reported association between prehospital time and outcomes was mixed in these studies with similar patient inclusion criteria.

### Prehospital time as a covariate

Prehospital time was used as a covariate in 38 of 96 (40%) full-text articles reviewed. For example, Pakkanen et al. evaluated the differences in outcomes in severe TBI patients based on the exposure of a paramedic-staffed response unit versus a physician-staffed model [73]. Other examples of the use of prehospital time as a covariate were among studies with prehospital interventions as a primary exposure (e.g., Chiang, et al. [46]).

### Prehospital time as an outcome

Prehospital time was used as an outcome measure in 10 (10%) studies [2, 61, 62, 74, 75, 83, 85, 88, 94, 98]. Four of these studies evaluated the time resultant from one of the following independent factors: prehospital endotracheal intubation, chest tube insertion, needle thoracostomy, tourniquet application, cricothyroidotomy, and advanced cardiac life support [61, 74, 75, 83]. For instance, Haltmeier et al. evaluated outcomes based on prehospital intubation in severe TBI patients (due to blunt trauma), comparing those to outcomes in patients that were not intubated in the prehospital setting. They found that there were associations between prehospital intubation and longer scene times (median 9 vs. 8 min *p* < 0.001), transport times (median 26 vs. 19 min, *p* < 0.001), days on a ventilator (mean 7.3 vs. 6.9, *p* = 0.006), ICU (median 6 vs 5 days, *p* < 0.001) and hospital length of stay (median 10 vs 9 days, *p* < 0.001), and higher in-hospital mortality (31.4 vs. 27.5%, *p* < 0.001) [61]. Meanwhile, three articles (corresponding to two research studies) investigated the effect on prehospital time due to initiation of prehospital plasma infusion and tranexamic acid (TXA) administration [85, 88, 89]. Lastly, three studies looked at prehospital time, measured as dispatch time to definitive care, as an outcome resultant from different system-based variables, including trauma “deserts” in an urban area [2], a physician-staffed vs paramedic-staffed regional rotary wing aeromedical (helicopter) EMS system [62], and indirect vs direct transfer of TBI patients [94]. Of note, the article by Hesselfeldt et al. was not primarily a direct versus indirect transfer investigation, but the need for secondary transfer to a tertiary trauma center from an outside facility was listed as an outcome.

#### Level of evidence

A vast majority (90, 94%) of full-text studies reviewed were observational and had corresponding “low” levels of evidence, per the GRADE criteria. There were few articles (19, 20%) that reached a “moderate” or “high” level of evidence based on large sample sizes, more rigorous study designs (e.g., interventional trials), and/or the ability to compare randomized interventional versus control arms. Full article summaries are available in Additional file 2. The articles with the largest numbers of enrolled subjects were derived from registry data from 3 main sources: the National Trauma Data Bank (NTDB) (e.g., [45]), the Department of Defense Trauma Registry (e.g., [73]), Germany’s Trauma Register DGU (e.g., [63]), or a regionally developed trauma registry (e.g., [32]).

## Discussion

Trauma continues to be a leading and growing cause of morbidity and mortality across the world. EMS systems provide the earliest opportunity for the trauma care system to initiate resuscitation and rapidly deliver patients to definitive care facilities. Prehospital trauma care and priorities are time-driven, so it is necessary to understand the relationship between time and outcomes to help identify opportunities to optimize prehospital care and improve trauma outcomes. Yet, experts state there is an inadequate evidence base to support EMS practice [113]. Our scoping review specifically assessed the types of published studies regarding the effect of prehospital time on trauma outcomes.

We identified 96 relevant articles and several key trends. First, we found a disproportionate minority (8%) of articles representing studies from LMICs, despite that over 90% of the global burden of injury originates from LMICs. Second, in-hospital mortality measured late in the clinical course, often at 30 days, was the most commonly used primary outcome measure, notwithstanding that these studies were prehospital-focused. For secondary outcomes, many studies measured length of stay (a process indicator) and only a minority of studies reported morbidity measures (e.g., organ failure). Third, the preponderance of studies was observational in design, many of which used trauma registries as the data source. Interventional prehospital trauma studies on this topic were rare. Last, studies primarily assessing the association of prehospital time and in-hospital mortality reported mixed (i.e., positive, negative, and neutral) associations, with conflicting conclusions [28, 30, 36, 40, 41, 56, 65, 68, 70, 77, 114].

Even though most of the trauma morbidity and mortality across the world arises from LMICs, and the fact that more than half of deaths in LMICs can be treated with prehospital and emergency care, LMICs are significantly underrepresented in this cohort of studies [13, 115] This finding supports prior statements by the World Health Organization that prehospital emergency care in LMICs is a neglected area of research. The reasons are multifactorial, likely due to a combination of limited in-country research resources, relative paucity of formal EMS systems, limited prehospital research expertise, and a hospital-centric focus on trauma outcomes in LMICs. Research from LMICs may help fill important scientific gaps. First, strong and consistent trends between time and outcomes may be found in lower income settings because higher trauma caseloads may yield higher sample sizes and fewer resuscitative interventions may limit confounding factors. Second, a large criticism of prehospital trauma studies in HICs, supported by findings in our scoping review, is that the majority are conducted in urban trauma systems with short (< 30 min) prehospital times which is not reflective of the longer times to definitive care experienced in the rest of the world. Hence, prehospital trauma research from LMICs may help fill the evidence gap on outcomes from prolonged care.

In-hospital mortality, often at 30 days, was the most commonly used trauma outcome. However, the median time from admission to hemorrhagic death is 2.0 to 2.6 h, according to several higher income country urban studies [116]. Consequently, military and civilian experts have urged the use of earlier time points, especially in resuscitation studies of time-sensitive, emergent injuries such as hemorrhagic shock [116]. Prehospital resuscitation and ambulance transport occur relatively early in the overall spectrum of a patient’s care and more likely to be reflected in proximal time points, within 1 to 7 days [116]. Longer term outcomes (e.g., 30-day mortality or hospital survival) are more likely to reflect the effects of on-going hospital care. Twenty-eight- and 30-day mortality have historically been a standard in hospital-based trauma research, which is beneficial by allowing comparisons of outcomes among studies. We also noted that few studies evaluated physiologic-based secondary outcomes, specifically single or multi-organ failure (MOF). MOF is a significant cause of post-injury morbidity and mortality and is impacted by early resuscitation [117]. MOF often starts around day 3 after injury and often peaks around day 7 [118]. Yet, we found a paucity of studies assessing MOF. We postulate that conducting prehospital trauma studies assessing MOF outcomes is relatively complex, as it requires the meticulous merging of prehospital data with in-hospital laboratory and clinical information, which is cost- and resource-prohibitive for most researchers, especially those without substantive research grants or infrastructure. Instead of physiologic outcomes, we found that many studies assessed secondary outcomes using process indicators (e.g., length of stay and mechanical ventilation days). While helpful, these are health system process indicators which limit comparability and generalizability of findings. TBI-focused studies often reported functional outcome measures assessed farthest from the date of injury, which is expected as neurologic outcomes usually evolve over weeks to months (e.g., Glasgow Outcomes Score at 6 months).

The majority of studies we reviewed were observational (mostly retrospective) in design. Prospective and interventional studies, often more complex and expensive to conduct, comprise the minority of all trauma research studies, and our scoping review noted this same trend reported in prior literature [119]. We found four prehospital trauma clinical trials corresponding to six articles, all related to administration of TXA and blood products to improve outcomes. Clinical trials in trauma are particularly challenging, considering the unpredictable nature of trauma which adds to the logistic and clinical difficulties [119]. The addition of the prehospital context further complicates the regulatory and practical aspects of trauma trials, partly explaining why prehospital trauma trials are especially rare. Hurdles encountered by prehospital trauma interventional studies include regulatory issues, informed consent, practitioner compliance, standardizing delivery of interventions, and EMS protocols that may conflict with trial protocols [119, 120]. We also found that a large proportion of observational studies were based upon trauma registry data. Most trauma registries are primarily developed to inform trauma quality improvement and for benchmarking care, as opposed to research [121]. Interestingly, the registry-based studies we reviewed often had a slightly higher level of evidence than non-registry based studies, likely resulting from larger sample sizes, use of well-defined and standardized data, and ability to control for relevant variables in statistical modeling [39]. An additional benefit of trauma registries is that they may represent larger and more diverse populations (e.g., state-based or regional registries), and conclusions drawn may better inform regional trauma system design, practices, and protocols. We do acknowledge that implementing trauma registries is challenging, especially in resource-constrained settings. There are limitations in registries even in higher-income settings, including variability in quality of data, consistent data collection, and difficulties in standardization of data, all of which would require mitigation if implemented in the LMIC setting [122]. A recent scoping review found 28 articles that reported challenges implementing trauma registries in LMICs, with the most significant barriers being ensuring data quality, lack of resources, inadequate prehospital care, and difficulty with administrative duties and hospital organization [121].

Last, there were conflicting results regarding the relationship between prehospital time and patient outcomes, especially mortality. As a scoping review, we did not quantitatively explore this; however, we do offer several possible explanations for this observation. First, trauma is a heterogeneous group of diseases, yet most studies we reviewed included all-comer (undifferentiated) trauma patients and often grouped patients by penetrating vs blunt injury. While important, mechanism of injury alone is inadequate to separate distinct physiologic subgroups of injuries (e.g., hemorrhagic shock vs tension pneumothorax vs TBI), which have competing physiologic derangements and resuscitative priorities. Accurate subgrouping by specific injuries may require hospital-based diagnoses, which adds complexity to prehospital study design and may deter investigators. Second, specific prehospital time intervals were often, but not always, reported, except for a minority of studies that controlled for the effect of response, scene, or transport durations on outcomes which may have caused conflicting findings across studies. Third, we found no studies that controlled for outcomes based on traumatic conditions, or body parts injured, that EMS practitioners can directly intervene upon to significantly influence patient outcomes. For example, limb amputations are directly intervenable by prehospital tourniquet application, whereas directly controlling abdominal hemorrhage is non-achievable by EMS practitioners. However, many studies we reviewed included both populations within the category of “hemorrhage,” which may help explain why some studies showed no benefit of EMS interventions, despite time, on hemorrhagic outcomes. Last, specific body parts or mechanism of injury was not assessed by many studies which may render the interpretation of results to be challenging considering the heterogeneity in trauma. We should note that most studies of undifferentiated patients performed subgroup analyses of blunt versus penetrating injuries, or head versus non-head injuries—while commendable, this approach is likely still inadequate considering the heterogeneity of injuries within subgroups. The notable exceptions were TBI and a few studies on torso injuries, which excluded cases with irrelevantly injured body parts.

Based on these findings, we offer several recommendations. Foremost, additional studies are needed to further investigate the effect of prehospital time and resuscitative interventions at shorter end-points (e.g., 72 h or 1 week) post-injury. Such approaches may better elucidate the specific impact of time and interventions on patient outcomes attributable to prehospital trauma care. Additionally, studies should place a heavier focus on morbidity measures (e.g., organ failure scores), especially via prehospital interventional trials, which can be more appropriately designed to assess causation of early prehospital interventions on hospital morbidity outcomes such as organ failure. Finally, there appears to be great need and potential benefit from conducting more prehospital trauma studies in LMICs, especially settings with high-prevalence and prolonged durations of care, which may more equitably address the worldwide burden of trauma—we recognize there are substantive challenges with resources and expertise that need to be overcome to accomplish this.

## Limitations

Searches in this scoping review were limited to more contemporary studies published between 2009 and 2019. Expanding search criteria to a wider time frame would have yielded a more comprehensive list of articles, though this would have challenged the relevance of the review due to the inclusion of aged studies. Another limitation is that we excluded articles solely focusing on special trauma sub-populations (i.e., incarcerated, pediatric, and pregnant patients) and certain injury patterns (i.e., electrocution and drownings). While methodologically beneficial to focus this work, our findings are less relevant to less common trauma populations and uncommon mechanisms of injury. We also limited our search to English language studies which likely limited our yield, given the worldwide focus, but was methodologically important to the English-speaking authors’ ability to evaluate the rigor and depth of reviews. Last, as a scoping review, we did not conduct a quantitative synthesis of study data, statistical techniques, or analytic limitations.

## Conclusion

Our scoping review evaluated 96 articles published on the relationship of prehospital time and in-hospital outcomes. Nearly all were observational in design, in which prehospital time was often used as a key exposure with in-hospital mortality, at 30 days, as a primary outcome. Relatively few studies were available from LMICs, despite LMICs contributing the largest share of injury morbidity and mortality globally. Trauma registries provided a robust data set for evaluation in many higher quality studies and would be a valuable tool in future international, prehospital trauma research in resource-limited settings. We recommend more interventional prehospital trials, which use short-term trauma outcomes to better reflect the effect of prehospital time and interventions, with substantively more investigations needed in LMICs. We encourage that future studies include more specific morbidity outcome measures, such as multi-organ dysfunction, in addition to process indicators.

## Supplementary Information

**Additional file 1:.** Search terms and syntax

**Additional file 2:.** Article Summaries

## Data Availability

All data generated or analyzed during this study are included in this published article and its supplementary information files.
