# Supplementary figures and images for: A scoping review of worldwide studies evaluating the effects of prehospital time on trauma outcomes
Source: Int J Emerg Med. 2020 Dec 9;13:64. doi: 10.1186/s12245-020-00324-7 (PMC7724615; doi:10.1186/s12245-020-00324-7)

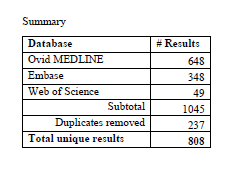

Supplement: Supplementary file 1 — Additional file 1:. Search terms and syntax [file 12245_2020_324_MOESM1_ESM.zip › Add 2.1.PNG]

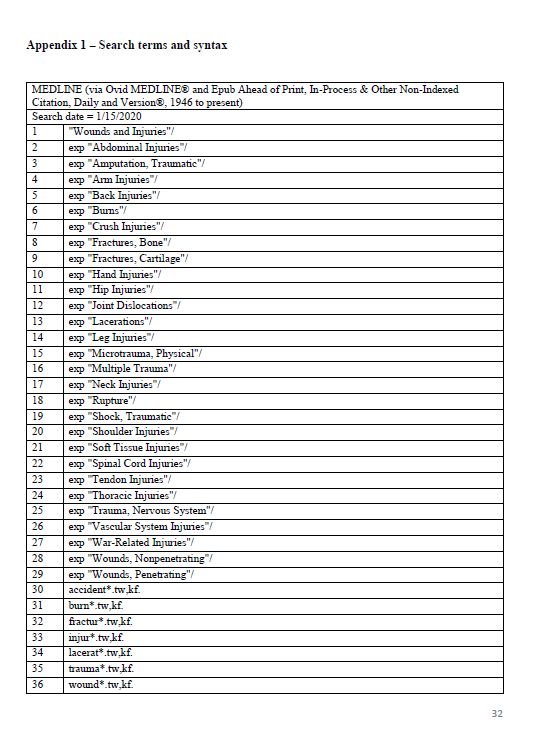

Supplement: Supplementary file 1 — Additional file 1:. Search terms and syntax [file 12245_2020_324_MOESM1_ESM.zip › Add1.PNG]

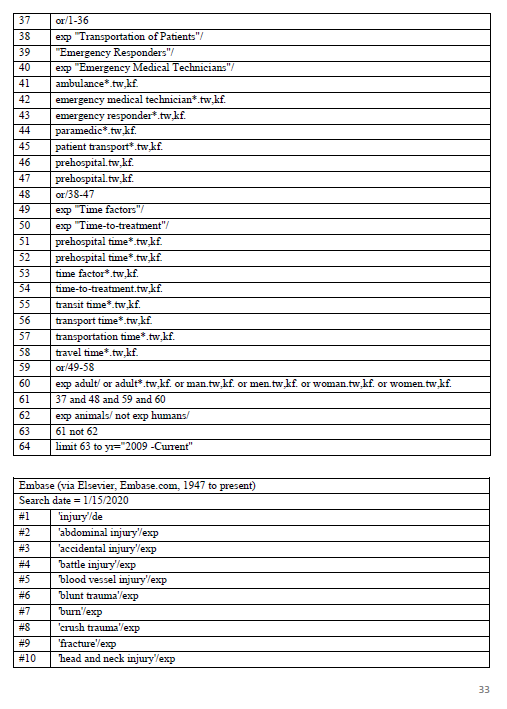

Supplement: Supplementary file 1 — Additional file 1:. Search terms and syntax [file 12245_2020_324_MOESM1_ESM.zip › add2.PNG]

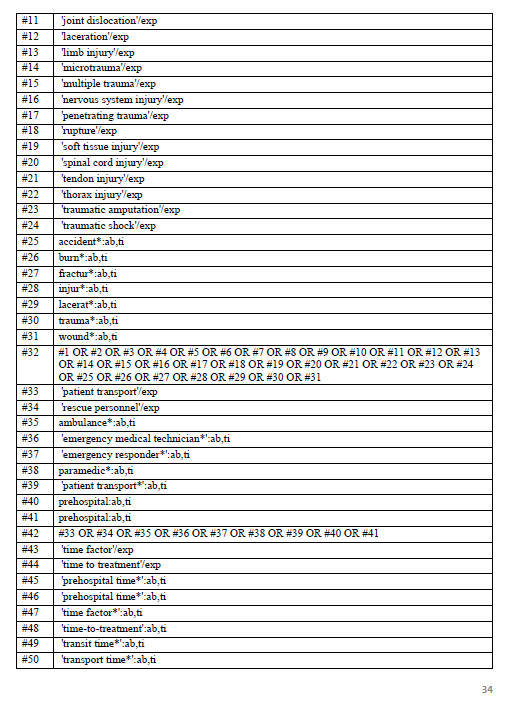

Supplement: Supplementary file 1 — Additional file 1:. Search terms and syntax [file 12245_2020_324_MOESM1_ESM.zip › add3.PNG]

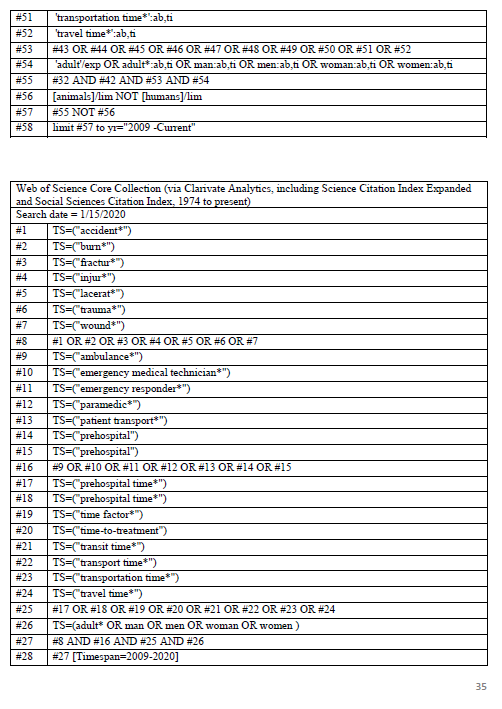

Supplement: Supplementary file 1 — Additional file 1:. Search terms and syntax [file 12245_2020_324_MOESM1_ESM.zip › add4.PNG]

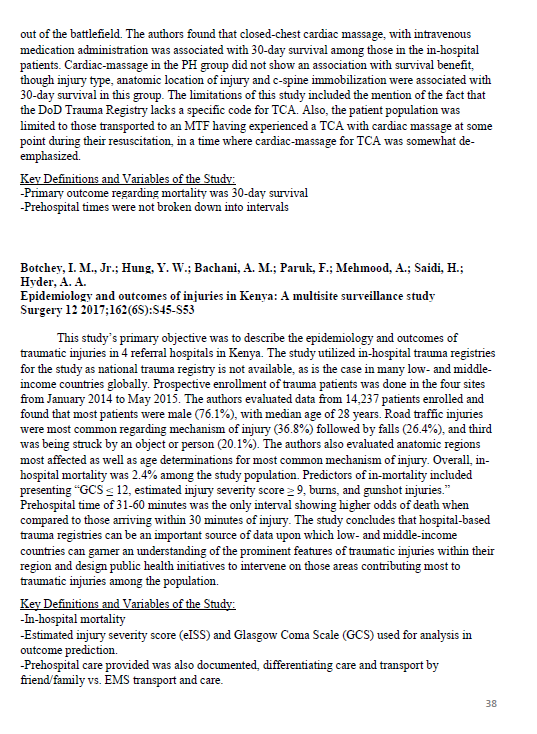

Supplement: Supplementary file 2 — Additional file 2:. Article Summaries [file 12245_2020_324_MOESM2_ESM.zip › Addfile2.1.PNG]

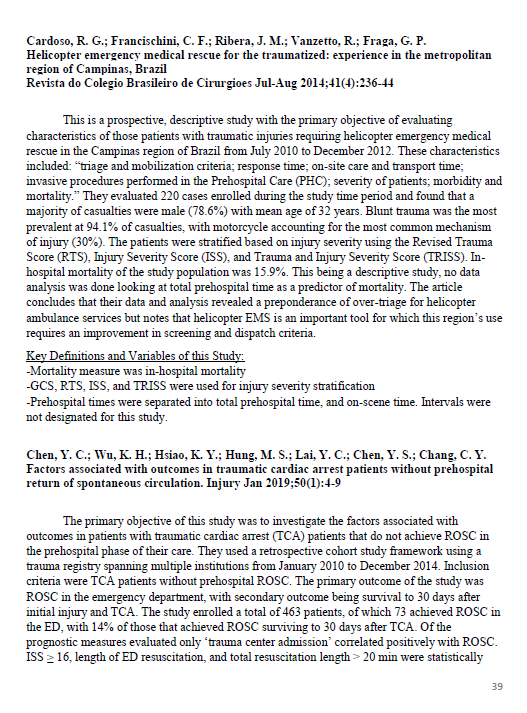

Supplement: Supplementary file 2 — Additional file 2:. Article Summaries [file 12245_2020_324_MOESM2_ESM.zip › Addfile2.2.PNG]

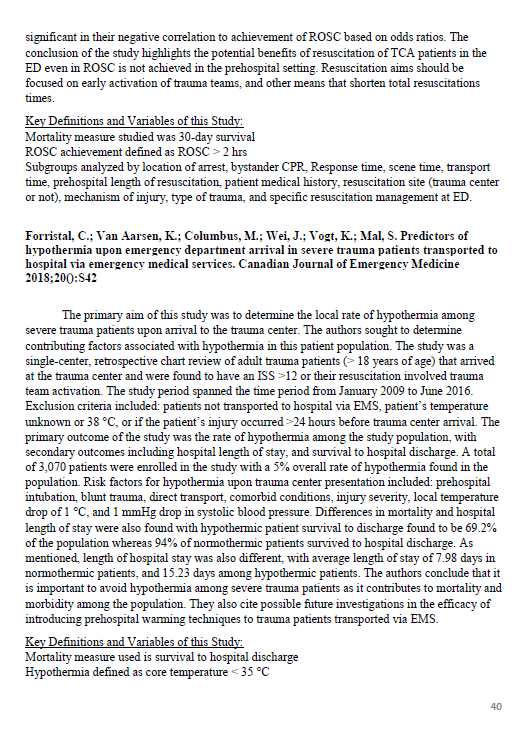

Supplement: Supplementary file 2 — Additional file 2:. Article Summaries [file 12245_2020_324_MOESM2_ESM.zip › Addfile2.3.PNG]

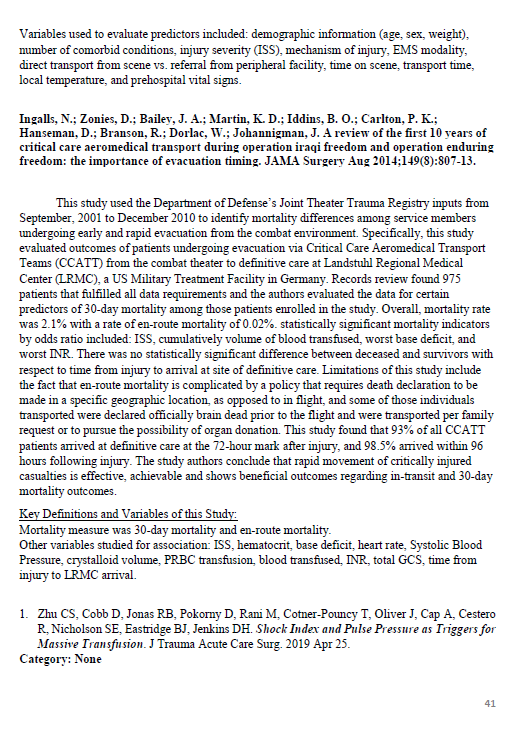

Supplement: Supplementary file 2 — Additional file 2:. Article Summaries [file 12245_2020_324_MOESM2_ESM.zip › Addfile2.4.PNG]

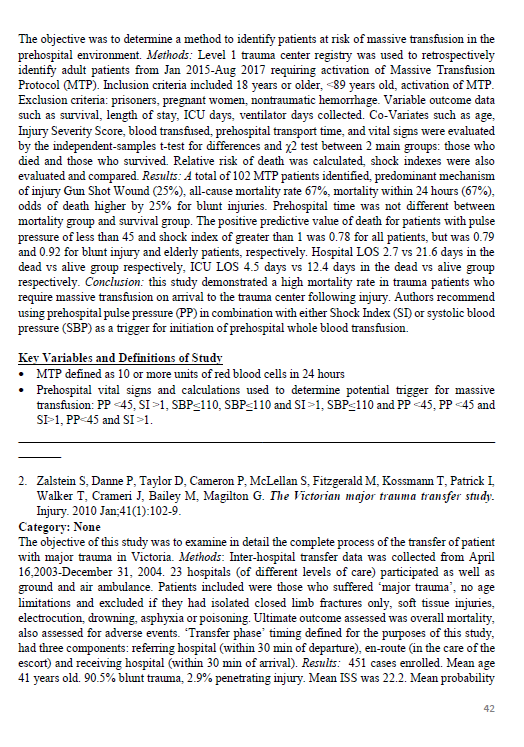

Supplement: Supplementary file 2 — Additional file 2:. Article Summaries [file 12245_2020_324_MOESM2_ESM.zip › Addfile2.5.PNG]

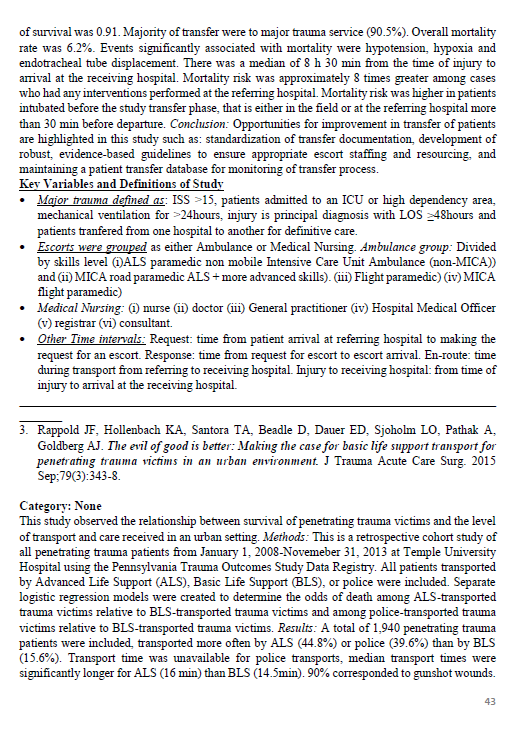

Supplement: Supplementary file 2 — Additional file 2:. Article Summaries [file 12245_2020_324_MOESM2_ESM.zip › addfile2.6.PNG]

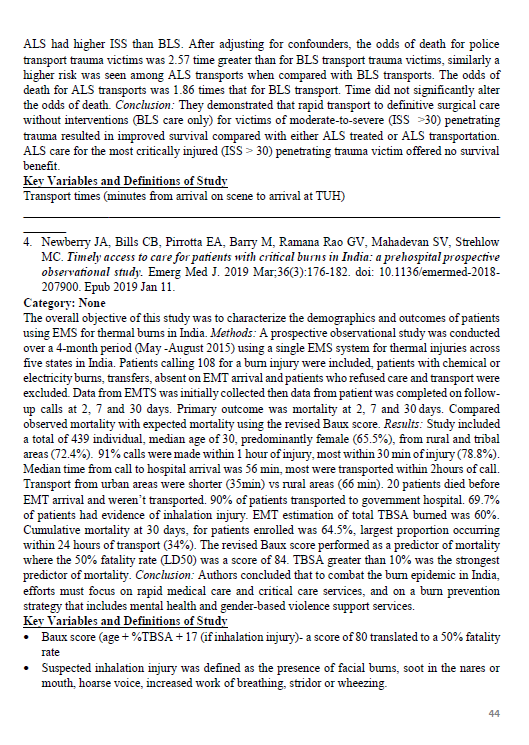

Supplement: Supplementary file 2 — Additional file 2:. Article Summaries [file 12245_2020_324_MOESM2_ESM.zip › Addfile2.7.PNG]

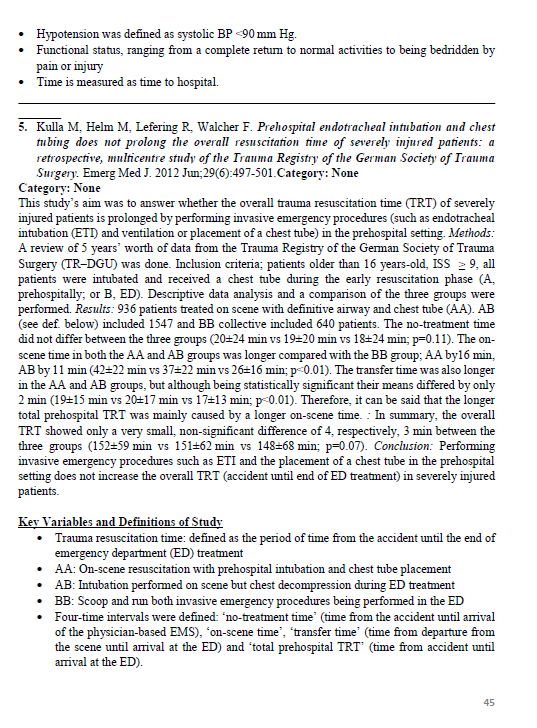

Supplement: Supplementary file 2 — Additional file 2:. Article Summaries [file 12245_2020_324_MOESM2_ESM.zip › Addfile2.8.PNG]

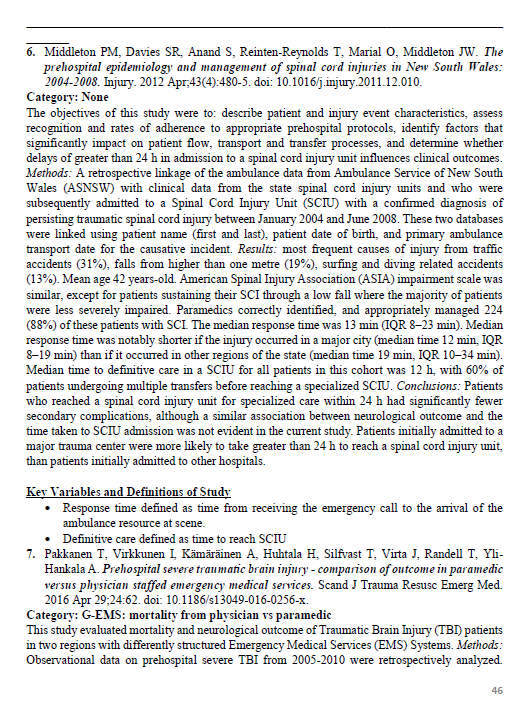

Supplement: Supplementary file 2 — Additional file 2:. Article Summaries [file 12245_2020_324_MOESM2_ESM.zip › Addfile2.9.PNG]

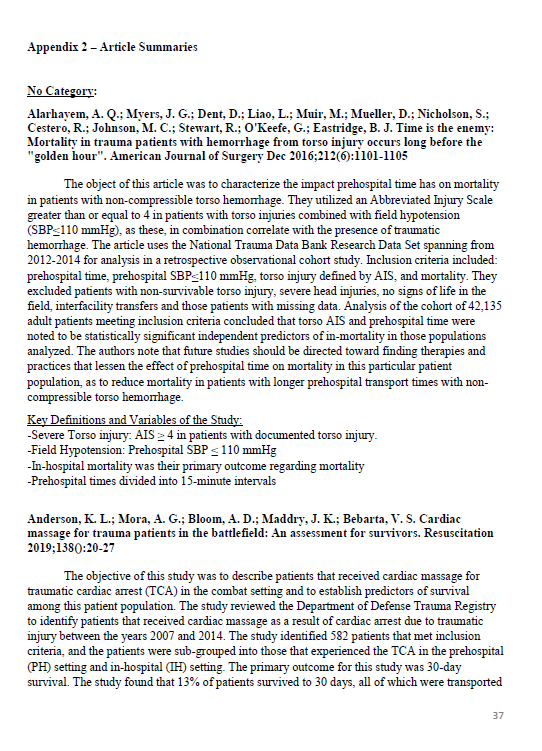

Supplement: Supplementary file 2 — Additional file 2:. Article Summaries [file 12245_2020_324_MOESM2_ESM.zip › Addfile2.PNG]

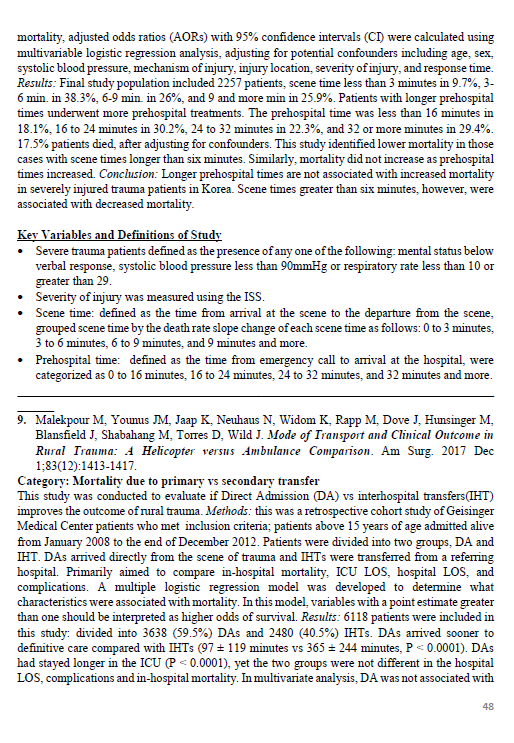

Supplement: Supplementary file 2 — Additional file 2:. Article Summaries [file 12245_2020_324_MOESM2_ESM.zip › Addfile3.1.PNG]

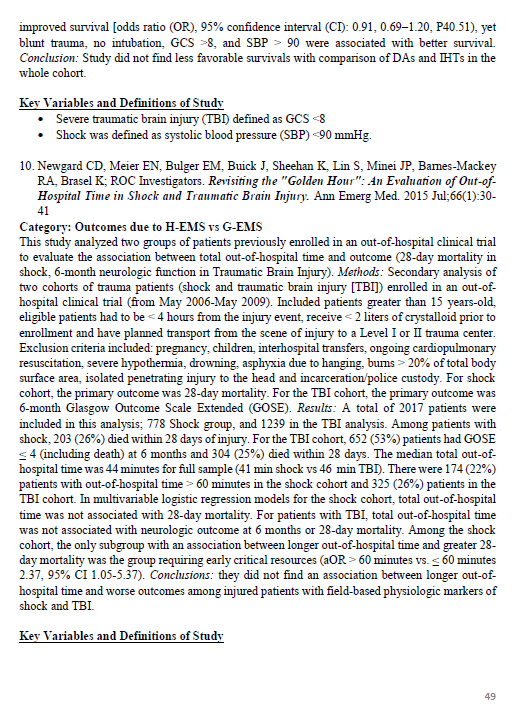

Supplement: Supplementary file 2 — Additional file 2:. Article Summaries [file 12245_2020_324_MOESM2_ESM.zip › Addfile3.2.PNG]

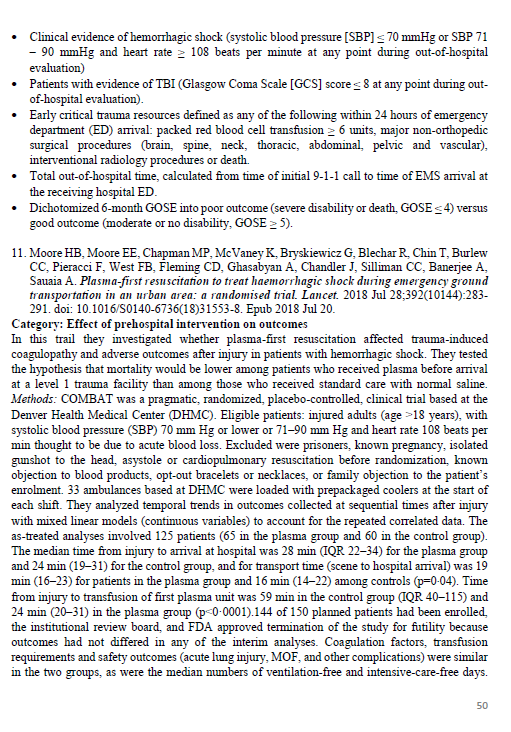

Supplement: Supplementary file 2 — Additional file 2:. Article Summaries [file 12245_2020_324_MOESM2_ESM.zip › Addfile3.3.PNG]

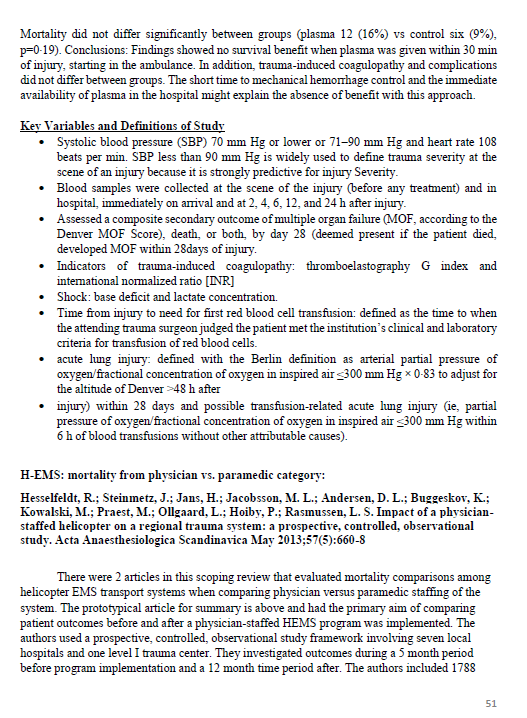

Supplement: Supplementary file 2 — Additional file 2:. Article Summaries [file 12245_2020_324_MOESM2_ESM.zip › Addfile3.4.PNG]

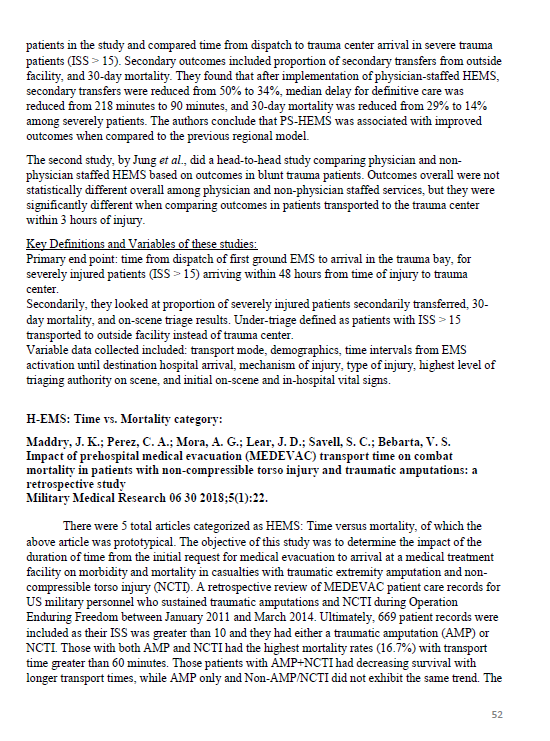

Supplement: Supplementary file 2 — Additional file 2:. Article Summaries [file 12245_2020_324_MOESM2_ESM.zip › Addfile3.5.PNG]

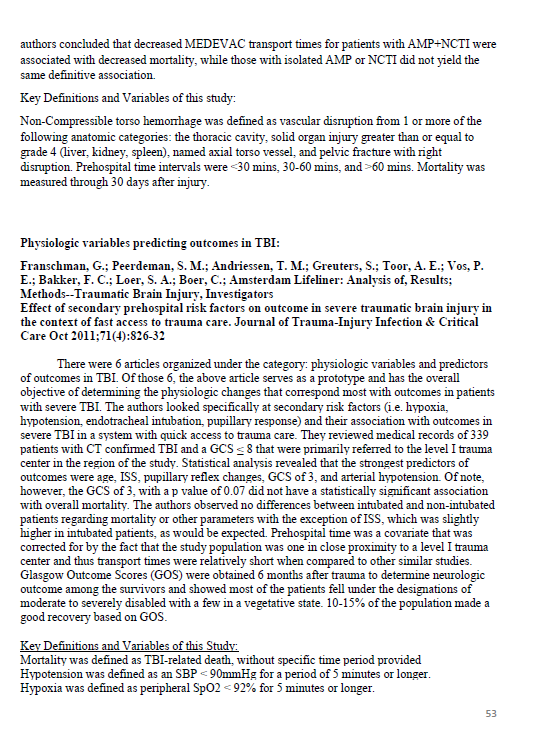

Supplement: Supplementary file 2 — Additional file 2:. Article Summaries [file 12245_2020_324_MOESM2_ESM.zip › Addfile3.6.PNG]

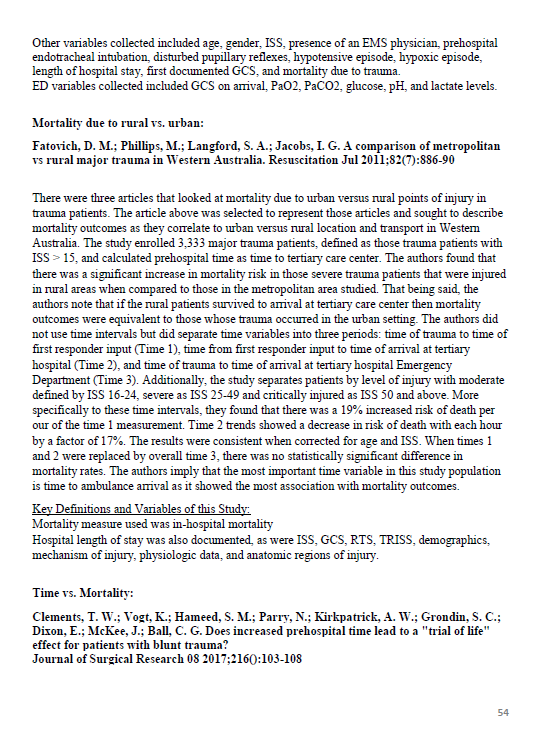

Supplement: Supplementary file 2 — Additional file 2:. Article Summaries [file 12245_2020_324_MOESM2_ESM.zip › Addfile3.7.PNG]

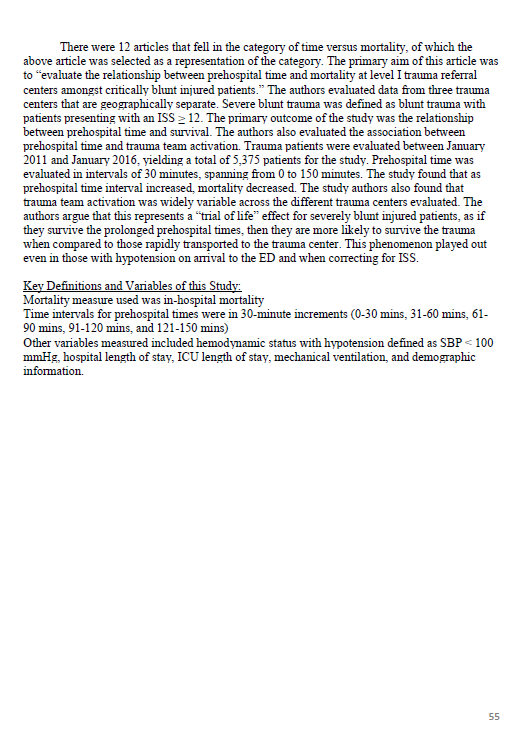

Supplement: Supplementary file 2 — Additional file 2:. Article Summaries [file 12245_2020_324_MOESM2_ESM.zip › Addfile3.8.PNG]

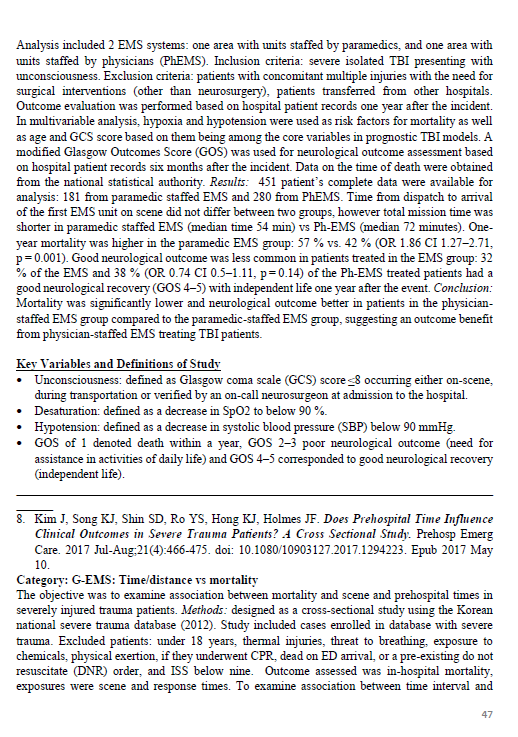

Supplement: Supplementary file 2 — Additional file 2:. Article Summaries [file 12245_2020_324_MOESM2_ESM.zip › Addfile3.PNG]
