# Supplementary material for: A scoping review of worldwide studies evaluating the effects of prehospital time on trauma outcomes
Source: Int J Emerg Med. 2020 Dec 9;13:64. doi: 10.1186/s12245-020-00324-7 (PMC7724615; doi:10.1186/s12245-020-00324-7)
Supplement: Supplementary file 2 — Additional file 2:. Article Summaries [file 12245_2020_324_MOESM2_ESM.zip › 12245_324.docx]

A scoping review of worldwide studies evaluating the effects of prehospital time on trauma outcomes

Alexander F. Bedard^1*,2^ [alexbedardmd@gmail.com](mailto:alexbedardmd@gmail.com),
Lina V. Mata^1^,
Chelsea Dymond^1,3^,
Fabio Moreira^4^,
Julia Dixon^1^,
Steven G. Schauer^5^,
Adit A. Ginde^1^,
Vikhyat Bebarta^1^,
Ernest E. Moore^1,6^,
Nee-Kofi Mould-Millman^1^

^1^University of Colorado
Anschutz Medical Campus, 13001 E 17th Place, Aurora, CO, 80045 USA

^2^ United States Air Force Medical Corps
7700 Arlington Boulevard, Falls Church, VA, 22042, USA

^3^Denver Health and Hospital Authority
777 Bannock St, Denver, CO, 80204, USA

^4^Western cape Government, Emergency Medical Services
**9 Wale Street, Cape Town, 8001,** South Africa

^5^US Army Institute of Surgical Research
3698 Chambers Rd., San Antonio, TX, 78234, USA

^6^Ernest E. Moore Shock Trauma Center at Denver Health
777 Bannock St, Denver, CO, 80204, USA

Abstract

Background

Annually, over 1 billion people sustain traumatic injuries, resulting in over 900,000 deaths in Africa and 6 million deaths globally. Timely response, intervention, and transportation in the prehospital setting reduce morbidity and mortality of trauma victims. Our objective was to describe the existing literature evaluating trauma morbidity and mortality outcomes as a function of prehospital care time to identify gaps in literature and inform future investigation.

Main body

We performed a scoping review of published literature in MEDLINE. Results were limited to English language publications from 2009 to 2020. Included articles reported trauma outcomes and prehospital time. We excluded case reports, reviews, systematic reviews, meta-analyses, comments, editorials, letters, and conference proceedings. In total, 808 articles were identified for title and abstract review. Of those, 96 articles met all inclusion criteria and were fully reviewed. Higher quality studies used data derived from trauma registries. There was a paucity of literature from studies in low- and middle-income countries (LMIC), with only 3 (3%) of articles explicitly including African populations. Mortality was an outcome measure in 93% of articles, predominantly defined as “in-hospital mortality” as opposed to mortality within a specified time frame. Prehospital time was most commonly assessed as crude time from EMS dispatch to arrival at a tertiary trauma center. Few studies evaluated physiologic morbidity outcomes such as multi-organ failure.

Conclusion

The existing literature disproportionately represents high-income settings and most commonly assessed in-hospital mortality as a function of crude prehospital time. Future studies should focus on how specific prehospital intervals impact morbidity outcomes (e.g., organ failure) and mortality at earlier time points (e.g., 3 or 7 days) to better reflect the effect of early prehospital resuscitation and transport. Trauma registries may be a tool to facilitate such research and may promote higher quality investigations in Africa and LMICs.

Keywords

Prehospital time
Trauma
Emergency medical services

1. Introduction

Trauma is a time-sensitive condition which accounts for approximately 12% of the global burden of disease [1]. Trauma has significant health and economic implications that disproportionally affect populations in low- and middle-income countries (LMICs). Globally, over one billion people sustain traumatic injuries, and over six million die annually [1]. The injury mortality rate in LMICs (9–12%) is double the proportion seen in high-income countries (5.5%), and up to 16% of all disabilities globally are attributed to injury [1–6]. The median cost of direct medical expenditures related to injury in a study of LMICs was 15% of GDP per capita annually [7]. Despite advances in trauma care and expansion of prevention programs, injury and associated mortality rates continue to rise [8–10]. The US Military, for example, has policies and training based on research in prolonged field care; however, trauma care research focused on the resource-limited setting is necessary to reduce civilian trauma mortality and disability in these regions [5, 11–13].

Timely prehospital care is key to improving outcomes in time-sensitive injuries [14, 15]. The concept of timely prehospital trauma care and rapid transport has been a mainstay in prehospital teaching since Dr. R. Adams Cowley identified the preponderance of mortality within 1 h of traumatic injury [16]. There are relatively few published studies reporting patient outcomes directly due to prehospital care, and even fewer studies assessing the independent effects of prehospital time on patient mortality [17–20].

The relationship between prehospital time and patient outcomes remains unclear and conflicting [21, 22]. A 2014 systematic review focused on prehospital time and outcomes, performed by Harmsen et al., included 20 level III evidence articles and concluded a decrease in odds of mortality for the undifferentiated trauma patient when response time or transfer time are shorter, but conversely, there was an increased odds of survival with increased on-scene time and total prehospital time [23]. This conflict may be explainable by the heterogeneous nature of prehospital care and broad spectrum of disease pathophysiology in trauma. Additionally, most prehospital studies are conducted in high-income country (HIC) urban settings with limited generalizability to rural and LMIC environments. In rural and LMIC settings, where prehospital times can be very prolonged, understanding the impact, efficacy, timing, and effect size of specific prehospital interventions could lead to improved patient outcomes. Findings from additional research can help identify opportunities to improve systems and care, ultimately optimizing morbidity and mortality outcomes [15]. Many published trauma studies include aspects of prehospital care and time; however, this is typically not the primary focus of the study.

We seek to appraise the global scope of contemporary trauma literature focused on prehospital time and trauma patient outcomes in order to identify trends and gaps, which can directly inform recommendations on areas in need of further research.

1. Methods

A scoping review of published literature was performed to critically appraise the relationship between trauma outcomes and prehospital time. A comprehensive literature search of MEDLINE, Embase, and Web of Science Core Collection databases was performed in January 2020. A combination of index terms and keywords including traumatic injury, prehospital time, and time to treatment were used to identify publications from 2009 to 2020 (Additional file 1: table 1). Results were limited to adult age group and exported to, and deduplicated in EndNote X9 (Clarivate Analytics, Philadelphia, PA). The Covidence systematic review software (Veritas Health Innovation, Melbourne, Australia) was used for screening and full text review.

For the first review, article abstracts were independently screened by two trained reviewers (AB, FM), blinded to each other’s reviews. Each reviewer read article titles and abstracts to determine if they satisfied inclusion criteria and to ensure they did not meet any exclusion criteria (see Table 1). Discrepant reviews of abstracts were adjudicated by a senior reviewer (NM).

Table 1 Screening and full-text article inclusion and exclusion criteria

| **Inclusion criteria** | **Exclusion criteria** |
| --- | --- |
| Trauma-focused study or report | No hospital outcomes (morbidity or mortality outcomes) |
| Time (as a covariate, key exposure, or outcome) | Electrocution injuries |
| EMS-focused study[*](#TF1) | Drowning injuries |
| Full text articles available | Focus on special populations (e.g., pediatrics, OB, incarcerated, psychiatric) |
| Adult patients | Field terminations (deceased on scene and not transported by EMS) |
| Published within the past 10 years | Case studies (or studies *N* < 50) |
| Articles written in English | Meta-analyses, systematic reviews, editorials, letters, and opinion pieces |
|  | Abstract only, no full manuscript published |

***Evidenced by EMS data, including vitals, transport modality, treatments, and/or transport time

Articles included after abstract review were divided between two reviewers (AB, LM) for a full text review and critical synthesis. The following key elements were assessed during each full text review: research questions, country, study design, injuries and populations studies, choice and definitions of independent and dependent variables, and level of evidence using GRADE criteria [24]. If any exclusion criteria were identified during full text review, the article was excluded with specific reason(s) provided (with approval from the senior reviewer). All included full text articles were coded into a summary table. Articles were grouped, based on common research categories, and one representative article from each category was summarized in a prose (paragraph) format. Articles not belonging to a specific category were individually summarized.

From the table of coded articles, key trends were descriptively reported using frequencies and percentages. Investigators independently appraised, then collectively discussed, all findings to reach consensus regarding key findings, conclusions, and recommendations which are presented qualitatively.

1. Results

We reviewed a total of 809 articles and included 96 after full text review (Fig. 1).

Study characteristics

Of 96 articles included, the overwhelming majority (90, 94%) were observational with a few (6, 6%) being interventional in design (Table 2) [25–30]. The six interventional studies evaluated the effects of prehospital blood product transfusion (plasma and packed red blood cells), and TXA administration on mortality, and used time (from injury to intervention) as a covariate. The largest proportion of articles originated from North America (42, 44%). Additional regions of origin included Europe (23, 24%), Asia (13, 14%), Australia (7, 7%), Africa (3, 3%), and South America (2, 2%). There were 6 (6%) articles of research simultaneously conducted in multiple geographic regions. We found 8 (8%) studies performed in LMICs, specifically Kenya, Malawi, Afghanistan, Iran, Iraq, and India. Of these, one study, conducted in Kenya, used a trauma registry as a data source [31]. The two studies in Afghanistan involve the US military patients only, as opposed to local trauma patients [32, 33]. The Iraqi studies, on the other hand, evaluated local prehospital trauma care and outcomes, aligning them more closely with other LMIC studies [34, 35].

Fig. 1 PRISMA [36] flowchart summarizing articles reviewed

Trauma mechanism and bodily injuries

Most studies included any trauma mechanism, commonly defined as external force to the body not including bites, stings, burns, or drownings. A specific mechanism of injury was stated in the inclusion criteria in relatively few studies, and mechanism was often either “blunt” [30, 37–39] or “penetrating” [40–42], though some did look at motor vehicle collisions as a specific mechanism [43, 44]. There were several studies that focused on isolated torso injuries [45, 46], but overall, the majority of articles (73, 76%) included any trauma mechanism to any body part. The notable exceptions were 17 (18%) studies of head-injured patients, which assessed the effect of prehospital interventions and/or prehospital time on neurologic outcomes [47–58].

- 1. Main outcomes

Mortality was a primary outcome in the majority (90, 94%) of articles. Other frequently used primary outcomes included neurologic decline among head-injured patients [47, 50, 55, 56, 59], duration of trauma resuscitation [60], and EMS response times [61]. For most studies, in-hospital mortality was the most frequently used mortality outcome measure and was most often defined as all-cause death during hospital admission. Several articles assessed mortality within a specified period of time, starting as early as prehospital or ED mortality, and as far out as 3-months post-injury [49], although follow-up periods beyond 3 months were less commonly used. In traumatic brain injury (TBI) and spinal cord injury studies, neurologically focused outcomes were often the primary outcome while mortality was a secondary outcome [49, 59]. In neurologic trauma studies, survivors’ outcomes were assessed at discharge or long after admission (often 3 to 6 months) using neurologic functional outcome measures (e.g., Glasgow Outcome Scale score).

- 1. Secondary outcomes

Secondary outcomes varied widely across articles, with the five most frequently used being hospital length of stay, intensive care unit (ICU) length of stay, days on mechanical ventilation, neurologic outcomes (most frequently Glasgow Outcome Scale), and EMS transport times (Table 2). Injury severity scoring measures were used in 73 (76%) articles to risk stratify and cohort similarly injured sub-groups of trauma patients, of which 54 (74%) used anatomic severity measures (injury severity score [ISS], abbreviated injury score [AIS], new injury severity score [NISS]); 3 (3%) used physiologic or hybrid scores (e.g., trauma injury severity score [TRISS]); and 17 (18%) used a combination of anatomic, physiologic, and/or hybrid scores (e.g., revised trauma score [RTS]). There were only a few studies that measured organ failure as a secondary outcome—four (4%) articles used multiple organ failure as a secondary outcome [27, 62–64] assessed by the Sequential Organ Failure Assessment (SOFA) score, and two (2%) studies specified acute renal failure as the organ failure outcome [25, 65].

- 1. Prehospital time as a key exposure

Prehospital time, the primary variable of interest of this scoping review, was used as a key exposure (independent variable) in 48 (50%) articles. Prehospital time was most commonly defined as crude time from EMS notification to hospital arrival time. A common objective of these studies was to assess the effect of prehospital time (total time, or seldom, time intervals) on pre- or in-hospital mortality. Studies reported mixed (negative, neutral, and positive) associations with mortality with shorter prehospital times. Fatovich et al., in their study of urban and rural trauma patients in Western Australia, found that the risk of death was two times higher among the rural population when compared to urban trauma patients (rural population experienced significantly longer times to definitive care with median times of 11.6 h versus 59 min, respectively). They also identified no difference in mortality outcomes when the rural trauma patient survived to admission to a tertiary trauma center, when compared to the urban trauma patient [66]. Bagher et al. found that on-scene time (median 17 min, IQR 11–23 min) and total prehospital time (median 35 min, IQR 27–46 min) had no associated effect on mortality among urban prehospital transports in Scandinavia [67]. Similarly, Brown et al. found no association between prehospital time “of one hour and 30-day mortality” (adjusted OR 1.1, 95% CI 0.71–1.69), but did find association between scene times and longer hospital lengths of stay, with each additional minute of on-scene time associated with 1.16 times longer length of hospital stay (95% CI 1.03–1.31) [68]. Finally, when total prehospital time was sub-divided into intervals (response time, scene time, and transport time), Brown et al. found that there was an association (OR 1.21; 95% CI 1.02–1.44, *p* = 0.03) between prolonged scene time and mortality, regardless of transport modality (air or ground) [69]. Therefore, the reported association between prehospital time and outcomes was mixed in these studies with similar patient inclusion criteria.

- 1. Prehospital time as a covariate

Prehospital time was used as a covariate in 38 of 96 (40%) full-text articles reviewed. For example, Pakkanen et al. evaluated the differences in outcomes in severe TBI patients based on the exposure of a paramedic-staffed response unit versus a physician-staffed model [56]. Other examples of the use of prehospital time as a covariate were among studies with prehospital interventions as a primary exposure (e.g., Chiang, et al. [70]).

- 1. Prehospital time as an outcome

Prehospital time was used as an outcome measure in 10 (10%) studies [2, 27, 28, 30, 52, 53, 57, 60, 61, 71]. Four of these studies evaluated the time resultant from one of the following independent factors: prehospital endotracheal intubation, chest tube insertion, needle thoracostomy, tourniquet application, cricothyroidotomy, and advanced cardiac life support [52, 53, 60, 71]. For instance, Haltmeier et al. evaluated outcomes based on prehospital intubation in severe TBI patients (due to blunt trauma), comparing those to outcomes in patients that were not intubated in the prehospital setting. They found that there were associations between prehospital intubation and longer scene times (median 9 vs. 8 min *p* < 0.001), transport times (median 26 vs. 19 min, *p* < 0.001), days on a ventilator (mean 7.3 vs. 6.9, *p* = 0.006), ICU (median 6 vs 5 days, *p* < 0.001) and hospital length of stay (median 10 vs 9 days, *p* < 0.001), and higher in-hospital mortality (31.4 vs. 27.5%, *p* < 0.001) [52]. Meanwhile, three articles (corresponding to two research studies) investigated the effect on prehospital time due to initiation of prehospital plasma infusion and tranexamic acid (TXA) administration [27, 28, 30]. Lastly, three studies looked at prehospital time, measured as dispatch time to definitive care, as an outcome resultant from different system-based variables, including trauma “deserts” in an urban area [2], a physician-staffed vs paramedic-staffed regional rotary wing aeromedical (helicopter) EMS system [61], and indirect vs direct transfer of TBI patients [57]. Of note, the article by Hesselfeldt et al. was not primarily a direct versus indirect transfer investigation, but the need for secondary transfer to a tertiary trauma center from an outside facility was listed as an outcome.

Level of evidence

A vast majority (90, 94%) of full-text studies reviewed were observational and had corresponding “low” levels of evidence, per the GRADE criteria. There were few articles (19, 20%) that reached a “moderate” or “high” level of evidence based on large sample sizes, more rigorous study designs (e.g., interventional trials), and/or the ability to compare randomized interventional versus control arms. Full article summaries are available in Additional file 2. The articles with the largest numbers of enrolled subjects were derived from registry data from 3 main sources: the National Trauma Data Bank (NTDB) (e.g., [72]), the Department of Defense Trauma Registry (e.g., [73]), Germany’s Trauma Register DGU (e.g., [63]), or a regionally developed trauma registry (e.g., [31]).

1. Discussion

Trauma continues to be a leading and growing cause of morbidity and mortality across the world. EMS systems provide the earliest opportunity for the trauma care system to initiate resuscitation and rapidly deliver patients to definitive care facilities. Prehospital trauma care and priorities are time-driven, so it is necessary to understand the relationship between time and outcomes to help identify opportunities to optimize prehospital care and improve trauma outcomes. Yet, experts state there is an inadequate evidence base to support EMS practice [74]. Our scoping review specifically assessed the types of published studies regarding the effect of prehospital time on trauma outcomes.

We identified 96 relevant articles and several key trends. First, we found a disproportionate minority (8%) of articles representing studies from LMICs, despite that over 90% of the global burden of injury originates from LMICs. Second, in-hospital mortality measured late in the clinical course, often at 30 days, was the most commonly used primary outcome measure, notwithstanding that these studies were prehospital-focused. For secondary outcomes, many studies measured length of stay (a process indicator) and only a minority of studies reported morbidity measures (e.g., organ failure). Third, the preponderance of studies was observational in design, many of which used trauma registries as the data source. Interventional prehospital trauma studies on this topic were rare. Last, studies primarily assessing the association of prehospital time and in-hospital mortality reported mixed (i.e., positive, negative, and neutral) associations, with conflicting conclusions [44, 67, 68, 75–82].

Even though most of the trauma morbidity and mortality across the world arises from LMICs, and the fact that more than half of deaths in LMICs can be treated with prehospital and emergency care, LMICs are significantly underrepresented in this cohort of studies [83, 84] This finding supports prior statements by the World Health Organization that prehospital emergency care in LMICs is a neglected area of research. The reasons are multifactorial, likely due to a combination of limited in-country research resources, relative paucity of formal EMS systems, limited prehospital research expertise, and a hospital-centric focus on trauma outcomes in LMICs. Research from LMICs may help fill important scientific gaps. First, strong and consistent trends between time and outcomes may be found in lower income settings because higher trauma caseloads may yield higher sample sizes and fewer resuscitative interventions may limit confounding factors. Second, a large criticism of prehospital trauma studies in HICs, supported by findings in our scoping review, is that the majority are conducted in urban trauma systems with short (< 30 min) prehospital times which is not reflective of the longer times to definitive care experienced in the rest of the world. Hence, prehospital trauma research from LMICs may help fill the evidence gap on outcomes from prolonged care.

In-hospital mortality, often at 30 days, was the most commonly used trauma outcome. However, the median time from admission to hemorrhagic death is 2.0 to 2.6 h, according to several higher income country urban studies [85]. Consequently, military and civilian experts have urged the use of earlier time points, especially in resuscitation studies of time-sensitive, emergent injuries such as hemorrhagic shock [85]. Prehospital resuscitation and ambulance transport occur relatively early in the overall spectrum of a patient’s care and more likely to be reflected in proximal time points, within 1 to 7 days [85]. Longer term outcomes (e.g., 30-day mortality or hospital survival) are more likely to reflect the effects of on-going hospital care. Twenty-eight- and 30-day mortality have historically been a standard in hospital-based trauma research, which is beneficial by allowing comparisons of outcomes among studies. We also noted that few studies evaluated physiologic-based secondary outcomes, specifically single or multi-organ failure (MOF). MOF is a significant cause of post-injury morbidity and mortality and is impacted by early resuscitation [86]. MOF often starts around day 3 after injury and often peaks around day 7 [87]. Yet, we found a paucity of studies assessing MOF. We postulate that conducting prehospital trauma studies assessing MOF outcomes is relatively complex, as it requires the meticulous merging of prehospital data with in-hospital laboratory and clinical information, which is cost- and resource-prohibitive for most researchers, especially those without substantive research grants or infrastructure. Instead of physiologic outcomes, we found that many studies assessed secondary outcomes using process indicators (e.g., length of stay and mechanical ventilation days). While helpful, these are health system process indicators which limit comparability and generalizability of findings. TBI-focused studies often reported functional outcome measures assessed farthest from the date of injury, which is expected as neurologic outcomes usually evolve over weeks to months (e.g., Glasgow Outcomes Score at 6 months).

The majority of studies we reviewed were observational (mostly retrospective) in design. Prospective and interventional studies, often more complex and expensive to conduct, comprise the minority of all trauma research studies, and our scoping review noted this same trend reported in prior literature [88]. We found four prehospital trauma clinical trials corresponding to six articles, all related to administration of TXA and blood products to improve outcomes. Clinical trials in trauma are particularly challenging, considering the unpredictable nature of trauma which adds to the logistic and clinical difficulties [88]. The addition of the prehospital context further complicates the regulatory and practical aspects of trauma trials, partly explaining why prehospital trauma trials are especially rare. Hurdles encountered by prehospital trauma interventional studies include regulatory issues, informed consent, practitioner compliance, standardizing delivery of interventions, and EMS protocols that may conflict with trial protocols [88, 89]. We also found that a large proportion of observational studies were based upon trauma registry data. Most trauma registries are primarily developed to inform trauma quality improvement and for benchmarking care, as opposed to research [90]. Interestingly, the registry-based studies we reviewed often had a slightly higher level of evidence than non-registry based studies, likely resulting from larger sample sizes, use of well-defined and standardized data, and ability to control for relevant variables in statistical modeling [91]. An additional benefit of trauma registries is that they may represent larger and more diverse populations (e.g., state-based or regional registries), and conclusions drawn may better inform regional trauma system design, practices, and protocols. We do acknowledge that implementing trauma registries is challenging, especially in resource-constrained settings. There are limitations in registries even in higher-income settings, including variability in quality of data, consistent data collection, and difficulties in standardization of data, all of which would require mitigation if implemented in the LMIC setting [92]. A recent scoping review found 28 articles that reported challenges implementing trauma registries in LMICs, with the most significant barriers being ensuring data quality, lack of resources, inadequate prehospital care, and difficulty with administrative duties and hospital organization [90].

Last, there were conflicting results regarding the relationship between prehospital time and patient outcomes, especially mortality. As a scoping review, we did not quantitatively explore this; however, we do offer several possible explanations for this observation. First, trauma is a heterogeneous group of diseases, yet most studies we reviewed included all-comer (undifferentiated) trauma patients and often grouped patients by penetrating vs blunt injury. While important, mechanism of injury alone is inadequate to separate distinct physiologic subgroups of injuries (e.g., hemorrhagic shock vs tension pneumothorax vs TBI), which have competing physiologic derangements and resuscitative priorities. Accurate subgrouping by specific injuries may require hospital-based diagnoses, which adds complexity to prehospital study design and may deter investigators. Second, specific prehospital time intervals were often, but not always, reported, except for a minority of studies that controlled for the effect of response, scene, or transport durations on outcomes which may have caused conflicting findings across studies. Third, we found no studies that controlled for outcomes based on traumatic conditions, or body parts injured, that EMS practitioners can directly intervene upon to significantly influence patient outcomes. For example, limb amputations are directly intervenable by prehospital tourniquet application, whereas directly controlling abdominal hemorrhage is non-achievable by EMS practitioners. However, many studies we reviewed included both populations within the category of “hemorrhage,” which may help explain why some studies showed no benefit of EMS interventions, despite time, on hemorrhagic outcomes. Last, specific body parts or mechanism of injury was not assessed by many studies which may render the interpretation of results to be challenging considering the heterogeneity in trauma. We should note that most studies of undifferentiated patients performed subgroup analyses of blunt versus penetrating injuries, or head versus non-head injuries—while commendable, this approach is likely still inadequate considering the heterogeneity of injuries within subgroups. The notable exceptions were TBI and a few studies on torso injuries, which excluded cases with irrelevantly injured body parts.

Based on these findings, we offer several recommendations. Foremost, additional studies are needed to further investigate the effect of prehospital time and resuscitative interventions at shorter end-points (e.g., 72 h or 1 week) post-injury. Such approaches may better elucidate the specific impact of time and interventions on patient outcomes attributable to prehospital trauma care. Additionally, studies should place a heavier focus on morbidity measures (e.g., organ failure scores), especially via prehospital interventional trials, which can be more appropriately designed to assess causation of early prehospital interventions on hospital morbidity outcomes such as organ failure. Finally, there appears to be great need and potential benefit from conducting more prehospital trauma studies in LMICs, especially settings with high-prevalence and prolonged durations of care, which may more equitably address the worldwide burden of trauma—we recognize there are substantive challenges with resources and expertise that need to be overcome to accomplish this.

1. Limitations

Searches in this scoping review were limited to more contemporary studies published between 2009 and 2019. Expanding search criteria to a wider time frame would have yielded a more comprehensive list of articles, though this would have challenged the relevance of the review due to the inclusion of aged studies. Another limitation is that we excluded articles solely focusing on special trauma sub-populations (i.e., incarcerated, pediatric, and pregnant patients) and certain injury patterns (i.e., electrocution and drownings). While methodologically beneficial to focus this work, our findings are less relevant to less common trauma populations and uncommon mechanisms of injury. We also limited our search to English language studies which likely limited our yield, given the worldwide focus, but was methodologically important to the English-speaking authors’ ability to evaluate the rigor and depth of reviews. Last, as a scoping review, we did not conduct a quantitative synthesis of study data, statistical techniques, or analytic limitations.

1. Conclusion

Our scoping review evaluated 96 articles published on the relationship of prehospital time and in-hospital outcomes. Nearly all were observational in design, in which prehospital time was often used as a key exposure with in-hospital mortality, at 30 days, as a primary outcome. Relatively few studies were available from LMICs, despite LMICs contributing the largest share of injury morbidity and mortality globally. Trauma registries provided a robust data set for evaluation in many higher quality studies and would be a valuable tool in future international, prehospital trauma research in resource-limited settings. We recommend more interventional prehospital trials, which use short-term trauma outcomes to better reflect the effect of prehospital time and interventions, with substantively more investigations needed in LMICs. We encourage that future studies include more specific morbidity outcome measures, such as multi-organ dysfunction, in addition to process indicators.

Acknowledgements

The authors would like to thank their friends and family for their support given the difficulty facing all of us during the COVID-19 pandemic.

Disclaimer

The views expressed in this article are those of the authors and do not reflect the official policy or position of the US Army Medical Department, Air Force Medical Service, Department of the Army, Department of the Air Force, Department of Defense, or the US Government.

Ethics approval and consent to participate

Since this is a scoping review, not involving human participants, data, or tissue, ethics review was not required.

Consent for publication

Since human subjects were not recruited or used in this study, consent for publication was not required.

Availability of data and materials

All data generated or analyzed during this study are included in this published article and its supplementary information files.

Competing interests

The authors of this study declare that they have no competing interests.

Funding

Funding for this study was provided by a US Department of Defense grant (#W81XWH1920055) awarded for a larger study investigating prolonged prehospital trauma care and resultant outcomes. Funding was used to pay a librarian for services rendered in the search for articles for review and to pay for article submission.

Authors’ contributions

AB was the primary author of the manuscript from first draft to final product. He reviewed all articles in the title and abstract screening and half of the articles provided for full text screening. He contributed to the generation of the table of articles, Fig. 1, and article summaries. LM reviewed half of the full text articles for inclusion, generated the PRISMA table for article review, contributed to the table of included studies, and helped in editing the manuscript. CD generated the published version of the table of articles and assisted in editing the manuscript. FM served as a secondary reviewer of titles and abstracts during that process of the scoping review, contributed to the table of articles, and assisted in editing the manuscript. JD, SS, AG, VB, and EM helped in editing the manuscript prior to publication. NM provided significant guidance and co-edited the manuscript and served as the final reviewer of the initial screening of articles and full text review. The authors read and approved the final manuscript.

1. Additional files

Additional file 1: Search terms and syntax

Additional file 2: Article Summaries

References

1. Haagsma JA, Graetz N, Bolliger I, Naghavi M, Higashi H, Mullany EC, et al. The global burden of injury: incidence, mortality, disability-adjusted life years and time trends from the Global Burden of Disease study 2013. Injury prevention. 2016;22(1):3-18.

2. Crandall M, Sharp D, Unger E, Straus D, Brasel K, Hsia R, et al. Trauma deserts: distance from a trauma center, transport times, and mortality from gunshot wounds in Chicago. Am J Public Health. 2013;103(6):1103-9.

3. Estimates W. Cause of death, by injury (% of total) - Low Income, Middle Income, High Income: The World Bank; 2019 [Available from: <https://data.worldbank.org/indicator/SH.DTH.INJR.ZS?locations=XM-XP-XD>.

4. Norman R, Matzopoulos R, Groenewald P, Bradshaw D. The high burden of injuries in South Africa. Bulletin of the World Health Organization. 2007;85:695-702.

5. Sasser SM, Varghese M, Joshipura M, Kellermann A. Preventing death and disability through the timely provision of prehospital trauma care. SciELO Public Health; 2006.

6. Sauaia A, Moore EE, Johnson JL, Chin TL, Banerjee A, Sperry JL, et al. Temporal trends of postinjury multiple-organ failure: still resource intensive, morbid, and lethal. J Trauma Acute Care Surg. 2014;76(3):582.

7. Wesson HKH, Boikhutso N, Bachani AM, Hofman KJ, Hyder AA. The cost of injury and trauma care in low- and middle-income countries: a review of economic evidence. Health Policy Plan. 2014;29(6):795-808.

8. Haagsma JA, Graetz N, Bolliger I, Naghavi M, Higashi H, Mullany EC, et al. The global burden of injury: incidence, mortality, disability-adjusted life years and time trends from the Global Burden of Disease study 2013. Inj Prev. 2016;22(1):3-18.

9. Krug EG, Organization WH. Injury: a leading cause of the global burden of disease. 1999.

10. Norman R, Matzopoulos R, Groenewald P, Bradshaw D. The high burden of injuries in South Africa. Bull World Health Organ. 2007;85(9):695-702.

11. Keenan S, Riesberg JC. Prolonged field care: beyond the “golden hour”. Wilderness Environ Med. 2017;28(2):S135-S9.

12. Kobusingye OC, Hyder AA, Bishai D, Joshipura M, Hicks ER, Mock C. Emergency medical services. Disease control priorities in developing countries. 2006;2(68):626-8.

13. Krug EG, Organization WH. Injury: a leading cause of the global burden of disease. World Health Organization; 1999.

14. Kobusingye OC, Hyder AA, Bishai D, Joshipura M, Hicks ER, Mock C. Emergency medical services. In: nd, Jamison DT, Breman JG, Measham AR, Alleyne G, Claeson M, et al., editors. Disease control priorities in developing countries. 2nd ed. Washington (DC) 2006.

15. Mould-Millman NK, Sasser SM, Wallis LA. Prehospital research in Sub-Saharan Africa: establishing research tenets. Acad Emerg Med. 2013;20(12):1304-9.

16. Rogers FB, Rittenhouse KJ, Gross BW. The golden hour in trauma: dogma or medical folklore? Injury. 2015;46(4):525-7.

17. Dinh MM, Bein K, Roncal S, Byrne CM, Petchell J, Brennan J. Redefining the golden hour for severe head injury in an urban setting: the effect of prehospital arrival times on patient outcomes. Injury. 2013;44(5):606-10.

18. Swaroop M, Straus DC, Agubuzu O, Esposito TJ, Schermer CR, Crandall ML. Pre-hospital transport times and survival for hypotensive patients with penetrating thoracic trauma. J Emerg Trauma Shock. 2013;6(1):16-20.

19. Gonzalez RP, Cummings GR, Phelan HA, Mulekar MS, Rodning CB. Does increased emergency medical services prehospital time affect patient mortality in rural motor vehicle crashes? A statewide analysis. The American Journal of Surgery. 2009;197(1):30-4.

20. Harmsen AMK, Giannakopoulos GF, Moerbeek PR, Jansma EP, Bonjer HJ, Bloemers FW. The influence of prehospital time on trauma patients outcome: a systematic review. Injury. 2015;46(4):602-9.

21. Gauss T, Ageron FX, Devaud ML, Debaty G, Travers S, Garrigue D, et al. Association of prehospital time to in-hospital trauma mortality in a physician-staffed emergency medicine system. JAMA Surg. 2019;25:25.

22. Möller A, Hunter L, Kurland L, van Hoving DJ. The association between hospital arrival time, transport method, prehospital time intervals, and in-hospital mortality in trauma patients presenting to Khayelitsha Hospital, Cape Town. African Journal of Emergency Medicine. 2018;8(3):89-94.

23. Harmsen A, Giannakopoulos G, Moerbeek P, Jansma E, Bonjer H, Bloemers F. The influence of prehospital time on trauma patients outcome: a systematic review. Injury. 2015;46(4):602-9.

24. Guyatt GH, Oxman AD, Schünemann HJ, Tugwell P, Knottnerus A. GRADE guidelines: a new series of articles in the Journal of Clinical Epidemiology. Journal of clinical epidemiology. 2011;64(4):380-2.

25. Kim BD, Zielinski MD, Jenkins DH, Schiller HJ, Berns KS, Zietlow SP. The effects of prehospital plasma on patients with injury: a prehospital plasma resuscitation. J Trauma Acute Care Surg. 2012;73(2 Suppl 1):S49-53.

26. Lyon RM, de Sausmarez E, McWhirter E, Wareham G, Nelson M, Matthies A, et al. Pre-hospital transfusion of packed red blood cells in 147 patients from a UK helicopter emergency medical service. Scand J Trauma Resusc Emerg Med. 2017;25(1):12.

27. Moore HB, Moore EE, Chapman MP, McVaney K, Bryskiewicz G, Blechar R, et al. Plasma-first resuscitation to treat haemorrhagic shock during emergency ground transportation in an urban area: a randomised trial. Lancet. 2018;392(10144):283-91.

28. Neeki MM, Dong F, Toy J, Vaezazizi R, Powell J, Wong D, et al. Tranexamic acid in civilian trauma care in the California Prehospital Antifibrinolytic Therapy Study. West J Emerg Med. 2018;19(6):977-86.

29. Pusateri AE, Moore EE, Moore HB, Le TD, Guyette FX, Chapman MP, et al. Association of prehospital plasma transfusion with survival in trauma patients with hemorrhagic shock when transport times are longer than 20 minutes: a post hoc analysis of the PAMPer and COMBAT clinical trials. JAMA Surg. 2020;155(2):e195085-e.

30. Reitz KM, Moore HB, Guyette FX, Sauaia A, Pusateri AE, Moore EE, et al. Prehospital plasma in injured patients is associated with survival principally in blunt injury: results from two randomized prehospital plasma trials. Journal of Trauma and Acute Care Surgery. 2020;88(1):33-41.

31. Botchey IM, Jr., Hung YW, Bachani AM, Paruk F, Mehmood A, Saidi H, et al. Epidemiology and outcomes of injuries in Kenya: a multisite surveillance study. Surgery. 2017;162(6S):S45-S53.

32. Kotwal RS, Howard JT, Orman JA, Tarpey BW, Bailey JA, Champion HR, et al. The effect of a golden hour policy on the morbidity and mortality of combat casualties. JAMA Surg. 2016;151(1):15-24.

33. Shackelford SA, Del Junco DJ, Powell-Dunford N, Mazuchowski EL, Howard JT, Kotwal RS, et al. Association of prehospital blood product transfusion during medical evacuation of combat casualties in Afghanistan with acute and 30-day survival. Jama. 2017;318(16):1581-91.

34. Murad MK, Issa DB, Mustafa FM, Hassan HO, Husum H. Prehospital trauma system reduces mortality in severe trauma: a controlled study of road traffic casualties in Iraq. Prehospital and disaster medicine. 2012;27(1):36.

35. Murad MK, Larsen S, Husum H. Prehospital trauma care reduces mortality. Ten-year results from a time-cohort and trauma audit study in Iraq. Scand J Trauma Resusc Emerg Med. 2012;20:13.

36. Moher D, Liberati A, Tetzlaff J, Altman DG, Group P. Preferred reporting items for systematic reviews and meta-analyses: the PRISMA statement. PLoS Med. 2009;6(7):e1000097.

37. Clements TW, Vogt K, Hameed SM, Parry N, Kirkpatrick AW, Grondin SC, et al. Does increased prehospital time lead to a “trial of life” effect for patients with blunt trauma? J Surg Res. 2017;216:103-8.

38. Jung K, Huh Y, Lee JC, Kim Y, Moon J, Youn SH, et al. Reduced mortality by physician-staffed HEMS dispatch for adult blunt trauma patients in Korea. J Korean Med Sci. 2016;31(10):1656-61.

39. Yeguiayan JM, Garrigue D, Binquet C, Jacquot C, Duranteau J, Martin C, et al. Medical pre-hospital management reduces mortality in severe blunt trauma: a prospective epidemiological study. Crit Care. 2011;15(1):R34.

40. Funder KS, Petersen JA, Steinmetz J. On-scene time and outcome after penetrating trauma: an observational study. Emerg Med J. 2011;28(9):797-801.

41. Rappold JF, Hollenbach KA, Santora TA, Beadle D, Dauer ED, Sjoholm LO, et al. The evil of good is better: making the case for basic life support transport for penetrating trauma victims in an urban environment. J Trauma Acute Care Surg. 2015;79(3):343-8.

42. Seamon MJ, Doane SM, Gaughan JP, Kulp H, D’Andrea AP, Pathak AS, et al. Prehospital interventions for penetrating trauma victims: a prospective comparison between advanced life support and basic life support. Injury. 2013;44(5):634-8.

43. Clark DE, Winchell RJ, Betensky RA. Estimating the effect of emergency care on early survival after traffic crashes. Accid Anal Prev. 2013;60:141-7.

44. Lovely R, Trecartin A, Ologun G, Johnston A, Svintozelskiy S, Vermeylen F, et al. Injury Severity Score alone predicts mortality when compared to EMS scene time and transport time for motor vehicle trauma patients who arrive alive to hospital. Traffic Inj Prev. 2018;19(sup2):S167-S8.

45. Alarhayem AQ, Myers JG, Dent D, Liao L, Muir M, Mueller D, et al. Time is the enemy: mortality in trauma patients with hemorrhage from torso injury occurs long before the “golden hour”. Am J Surg. 2016;212(6):1101-5.

46. Maddry JK, Perez CA, Mora AG, Lear JD, Savell SC, Bebarta VS. Impact of prehospital medical evacuation (MEDEVAC) transport time on combat mortality in patients with non-compressible torso injury and traumatic amputations: a retrospective study. Mil. 2018;5(1):22.

47. Berlot G, La Fata C, Bacer B, Biancardi B, Viviani M, Lucangelo U, et al. Influence of prehospital treatment on the outcome of patients with severe blunt traumatic brain injury: a single-centre study. Eur J Emerg Med. 2009;16(6):312-7.

48. Brazinova A, Majdan M, Leitgeb J, Trimmel H, Mauritz W, Austrian Working Group on Improvement of Early TBIC. Factors that may improve outcomes of early traumatic brain injury care: prospective multicenter study in Austria. Scand J Trauma Resusc Emerg Med. 2015;23:53.

49. Brorsson C, Rodling-Wahlstrom M, Olivecrona M, Koskinen LO, Naredi S. Severe traumatic brain injury: consequences of early adverse events. Acta Anaesthesiol Scand. 2011;55(8):944-51.

50. Franschman G, Peerdeman SM, Andriessen TM, Greuters S, Toor AE, Vos PE, et al. Effect of secondary prehospital risk factors on outcome in severe traumatic brain injury in the context of fast access to trauma care. J Trauma. 2011;71(4):826-32.

51. Fuller G, Woodford M, Lawrence T, Coats T, Lecky F. Do prolonged primary transport times for traumatic brain injury patients result in deteriorating physiology? A cohort study. Prehosp Emerg Care. 2014;18(1):60-7.

52. Haltmeier T, Benjamin E, Siboni S, Dilektasli E, Inaba K, Demetriades D. Prehospital intubation for isolated severe blunt traumatic brain injury: worse outcomes and higher mortality. Eur. 2017;43(6):731-9.

53. Lansom JD, Curtis K, Goldsmith H, Tzannes A. The effect of prehospital intubation on treatment times in patients with suspected traumatic brain injury. Air Med J. 2016;35(5):295-300.

54. Majidi S, Siddiq F, Qureshi AI. Prehospital neurologic deterioration is independent predictor of outcome in traumatic brain injury: analysis from National Trauma Data Bank. Am J Emerg Med. 2013;31(8):1215-9.

55. Newgard CD, Meier EN, Bulger EM, Buick J, Sheehan K, Lin S, et al. Revisiting the “Golden Hour”: an evaluation of out-of-hospital time in shock and traumatic brain injury. Ann Emerg Med. 2015;66(1):30-41, .e1-3.

56. Pakkanen T, Virkkunen I, Kamarainen A, Huhtala H, Silfvast T, Virta J, et al. Pre-hospital severe traumatic brain injury - comparison of outcome in paramedic versus physician staffed emergency medical services. Scand J Trauma Resusc Emerg Med. 2016;24:62.

57. Prabhakaran K, Petrone P, Lombardo G, Stoller C, Policastro A, Marini CP. Mortality rates of severe traumatic brain injury patients: impact of direct versus nondirect transfers. J Surg Res. 2017;219:66-71.

58. Spaite DW, Hu C, Bobrow BJ, Chikani V, Barnhart B, Gaither JB, et al. Association of out-of-hospital hypotension depth and duration with traumatic brain injury mortality. Ann Emerg Med. 2017;70(4):522-30.e1.

59. Foster NA, Elfenbein DM, Kelley W, Jr., Brown CR, Foley C, Scarborough JE, et al. Comparison of helicopter versus ground transport for the interfacility transport of isolated spinal injury. Spine J. 2014;14(7):1147-54.

60. Kulla M, Helm M, Lefering R, Walcher F. Prehospital endotracheal intubation and chest tubing does not prolong the overall resuscitation time of severely injured patients: a retrospective, multicentre study of the Trauma Registry of the German Society of Trauma Surgery. Emerg Med J. 2012;29(6):497-501.

61. Hesselfeldt R, Steinmetz J, Jans H, Jacobsson ML, Andersen DL, Buggeskov K, et al. Impact of a physician-staffed helicopter on a regional trauma system: a prospective, controlled, observational study. Acta Anaesthesiol Scand. 2013;57(5):660-8.

62. Andruszkow H, Lefering R, Frink M, Mommsen P, Zeckey C, Rahe K, et al. Survival benefit of helicopter emergency medical services compared to ground emergency medical services in traumatized patients. Crit Care. 2013;17(3):R124.

63. Hussmann B, Lefering R, Waydhas C, Touma A, Kauther MD, Ruchholtz S, et al. Does increased prehospital replacement volume lead to a poor clinical course and an increased mortality? A matched-pair analysis of 1896 patients of the Trauma Registry of the German Society for Trauma Surgery who were managed by an emergency doctor at the accident site. Injury. 2013;44(5):611-7.

64. Hussmann B, Schoeneberg C, Jungbluth P, Heuer M, Lefering R, Maek T, et al. Enhanced prehospital volume therapy does not lead to improved outcomes in severely injured patients with severe traumatic brain injury. BMC emerg. 2019;19(1):13.

65. Malekpour M, Younus JM, Jaap K, Neuhaus N, Widom K, Rapp M, et al. Mode of transport and clinical outcome in rural trauma: a helicopter versus ambulance comparison. Am Surg. 2017;83(12):1413-7.

66. Fatovich DM, Phillips M, Langford SA, Jacobs IG. A comparison of metropolitan vs rural major trauma in Western Australia. Resuscitation. 2011;82(7):886-90.

67. Bagher A, Todorova L, Andersson L, Wingren CJ, Ottosson A, Wangefjord S, et al. Analysis of pre-hospital rescue times on mortality in trauma patients in a Scandinavian urban setting. Trauma. 2017;19(1):28-34.

68. Brown E, Tohira H, Bailey P, Fatovich D, Pereira G, Finn J. Longer prehospital time was not associated with mortality in major trauma: a retrospective cohort study. Prehosp Emerg Care. 2019;23(4):527-37.

69. Brown JB, Rosengart MR, Forsythe RM, Reynolds BR, Gestring ML, Hallinan WM, et al. Not all prehospital time is equal: influence of scene time on mortality. J Trauma Acute Care Surg. 2016;81(1):93-100.

70. Chiang WC, Chen SY, Ko PC, Hsieh MJ, Wang HC, Huang EP, et al. Prehospital intravenous epinephrine may boost survival of patients with traumatic cardiac arrest: a retrospective cohort study. Scand J Trauma Resusc Emerg Med. 2015;23:102.

71. Meizoso JP, Valle EJ, Allen CJ, Ray JJ, Jouria JM, Teisch LF, et al. Decreased mortality after prehospital interventions in severely injured trauma patients. J Trauma Acute Care Surg. 2015;79(2):227-31.

72. Chen X, Guyette FX, Peitzman AB, Billiar TR, Sperry JL, Brown JB. Identifying patients with time-sensitive injuries: association of mortality with increasing prehospital time. Journal of trauma and acute care surgery. 2019;86(6):1015-22.

73. Kotwal RS, Scott LLF, Janak JC, Tarpey BW, Howard JT, Mazuchowski EL, et al. The effect of prehospital transport time, injury severity, and blood transfusion on survival of US military casualties in Iraq. J Trauma Acute Care Surg. 2018;85(1S Suppl 2):S112-S21.

74. Medicine Io. Emergency medical services: at the crossroads. Washington, DC: The National Academies Press; 2007. 310 p.

75. Borst GM, Davies SW, Waibel BH, Leonard KL, Rinehart SM, Newell MA, et al. When birds can’t fly: an analysis of interfacility ground transport using advanced life support when helicopter emergency medical service is unavailable. J Trauma Acute Care Surg. 2014;77(2):331-6; discussion 6-7.

76. Bulger EM, Guffey D, Guyette FX, MacDonald RD, Brasel K, Kerby JD, et al. Impact of prehospital mode of transport after severe injury: a multicenter evaluation from the Resuscitation Outcomes Consortium. J Trauma Acute Care Surg. 2012;72(3):567-73; discussion 73-5; quiz 803.

77. Byrne JP, Mann NC, Hoeft CJ, Buick J, Karanicolas P, Rizoli S, et al. The impact of short prehospital times on trauma center performance benchmarking: an ecologic study. J Trauma Acute Care Surg. 2016;80(4):586-94; discussion 94-6.

78. Chen X, Gestring ML, Rosengart MR, Billiar TR, Peitzman AB, Sperry JL, et al. Speed is not everything: identifying patients who may benefit from helicopter transport despite faster ground transport. J Trauma Acute Care Surg. 2018;84(4):549-57.

79. Fuller G, Lawrence T, Woodford M, Coats T, Lecky F. Emergency medical services interval and mortality in significant head injury: a retrospective cohort study. Eur J Emerg Med. 2015;22(1):42-8.

80. Ingalls N, Zonies D, Bailey JA, Martin KD, Iddins BO, Carlton PK, et al. A review of the first 10 years of critical care aeromedical transport during operation Iraqi freedom and operation enduring freedom: the importance of evacuation timing. JAMA Surg. 2014;149(8):807-13.

81. Kidher E, Krasopoulos G, Coats T, Charitou A, Magee P, Uppal R, et al. The effect of prehospital time related variables on mortality following severe thoracic trauma. Injury. 2012;43(9):1386-92.

82. Kim J, Song KJ, Shin SD, Ro YS, Hong KJ, Holmes JF. Does prehospital time influence clinical outcomes in severe trauma patients?: a cross sectional study. Prehosp Emerg Care. 2017;21(4):466-75.

83. Mould-Millman NK, Sasser SM, Wallis LA. Prehospital research in Sub-Saharan Africa: establishing research tenets. Acad Emerg Med. 2013;20(12):1304-9.

84. Organization WH. Governing body matters: key issues arising out of the seventy-second World Health Assembly and the 144th and 145th sessions of the WHO Executive Board. World Health Organization. Regional Office for South-East Asia; 2019.

85. Fox EE, Holcomb JB, Wade CE, Bulger EM, Tilley BC. Earlier endpoints are required for hemorrhagic shock trials among severely injured patients. Shock. 2017;47(5):567-73.

86. Dewar D, Moore FA, Moore EE, Balogh Z. Postinjury multiple organ failure. Injury. 2009;40(9):912-8.

87. Mukonkole S, Hunter L, Möller A, Mccaul M, Lahri S, Van Hoving D. A comparison of trauma scoring systems for trauma-related injuries presenting to a district-level urban public hospital in Western Cape, South Africa. S Afr J Surg. 2020;58(1):9-14.

88. Perry D, Griffin X, Parsons N, Costa M. Designing clinical trials in trauma surgery: overcoming research barriers. Bone & joint research. 2014;3(4):123-9.

89. Hargreaves K, Goodacre S, Mortimer P. Paramedic perceptions of the feasibility and practicalities of prehospital clinical trials: a questionnaire survey. Emerg Med J. 2014;31(6):499-504.

90. Bommakanti K, Feldhaus I, Motwani G, Dicker RA, Juillard C. Trauma registry implementation in low-and middle-income countries: challenges and opportunities. J Surg Res. 2018;223:72-86.

91. Brown JB, Stassen NA, Bankey PE, Sangosanya AT, Cheng JD, Gestring ML. Helicopters and the civilian trauma system: national utilization patterns demonstrate improved outcomes after traumatic injury. J Trauma. 2010;69(5):1030-4; discussion 4-6.

92. Zehtabchi S, Nishijima DK, McKay MP, Clay Mann N. Trauma registries: history, logistics, limitations, and contributions to emergency medicine research. Acad Emerg Med. 2011;18(6):637-43.

Table 2 Coded summaries of included full text articles

| **Article reference** | **Category** | **EMS System** | **Setting** | **Country** | **Design** | **Time** | **Primary Outcome** | **Secondary Outcome** | **Primary Exposure(s)** | **Grade** |
| --- | --- | --- | --- | --- | --- | --- | --- | --- | --- | --- |
| Aiolfi (2018) | Outcomes due to H-EMS vs G-EMS | Both | B | USA | O | Key exposure | In-hospital mortality | ICU LOS; hospital LOS | TBI; transport modality | Low |
| Al Thani (2014) | Effect of PH intervention on outcomes | Both | B | Qatar | O | Covariate | PH and in-hospital mortality | - | Trauma; intubation | Low |
| Alarhayem et al. [45] | Miscellaneous | Both | B | USA | O | Key exposure | In-hospital mortality | - | Non-compressible torso trauma; PHT | Low |
| Anderson (2019) | Miscellaneous | Both | C | Multiple | O | Key exposure | 30-day survival | Predictors of survival | Traumatic cardiac arrest | Low |
| Andruszkow et al. [62] | Outcomes due to H-EMS vs G-EMS | Both | B | Germany | O | Covariate | In-hospital mortality | Multiple organ dysfunction syndrome and/or sepsis | Transport modality | Low |
| Bagher et al. [67] | G-EMS: time/distance vs mortality | G-EMS | CU | Sweden | O | Key exposure | Mortality | - | Total PH time, on scene time, PH rescue times | Low |
| Berlot et al. [47] | Outcomes due to H-EMS vs G-EMS | Both | B | Italy | O | Key exposure | Survival to discharge neurologic disability | - | TBI; transport modality | Low |
| Borst et al. [75] | Outcomes due to H-EMS vs G-EMS | Both | B | USA | O | Key exposure | In-hospital mortality | - | Trauma center transfer; transport modality | Low |
| Boschini (2016) | Mortality due to primary vs secondary transfer | Both | B | Malawi | O | Covariate | In-hospital mortality | - | Primary versus secondary transfer to tertiary trauma center | Low |
| Botchey et al. [31] | Miscellaneous | Both | B | Kenya | O | Covariate | In-hospital mortality | - | Trauma | Low |
| Boudreau (2019) | Effect of PH intervention on outcomes | Air | CU | USA | O | Covariate | In-hospital mortality | VTE development | Trauma; PH TXA administration in H-EMS | Low |
| Brazinova et al. [48] | Physiologic variables predicting outcomes in TBI | Both | B | Austria | O | Covariate | In-hospital mortality | Favorable neurologic outcomes | TBI; recommended early interventions | Low |
| Brorsson et al. [49] | Physiologic variables predicting outcomes in TBI | Both | B | Sweden | O | Key exposure | Mortality at 3 months post injury | Neurologic outcomes based on Glasgow Outcome Scale | Severe TBI (GCS ≤ 8) | Very low |
| Brown et al. [68] | G-EMS: time/distance vs mortality | G-EMS | B | Australia | O | Key exposure | 30 day mortality | Hospital LOS for 30 day survivors | PH total time > 60 min; prolonged time intervals in either response; on-scene; transport; total | Low |
| Brown et al. [69] | Outcomes due to H-EMS vs G-EMS | Air | B | Australia | O | Key exposure | In-hospital mortality | - | H-EMS transport; time intervals | Low |
| Brown et al. [69] | G-EMS: Time/distance vs mortality | Both | B | USA | O | Key exposure | In-hospital mortality | - | Total PH time > 20 min | Low |
| Brown (2011) | Outcomes due to H-EMS vs G-EMS | Both | B | USA | O | Covariate | Survival to hospital discharge | Hospital LOS; ICU admission; mechanical ventilation; emergent operations | Interfacility transfer of trauma patients HEMS and GEMS | Low |
| Brown et al. [91] | Outcomes due to H-EMS vs G-EMS | Both | B | USA | O | Covariate | Survival to hospital discharge | Hospital resource utilization; ICU admission; mechanical ventilation | HEMS vs GEMS transport for trauma patients | Low |
| Bulger et al. [76] | Outcomes due to H-EMS vs G-EMS | Both | B | USA | O | Key exposure | 24 h survival | Survival to 28 days; 6-month GOS | Transport modality; hypovolemic shock; severe TBI | Low |
| Byrne et al. [77] | G-EMS: time/distance vs mortality | G-EMS | CU | USA | O | Key exposure | ED mortality | In-hospital mortality | PH time | Low |
| Cardoso (2014) | Miscellaneous | Air | B | Brazil | O | Covariate | In-hospital mortality | Hospital length of stay | HEMS transport for trauma | Low |
| Chen (2014) | Effect of PH intervention on outcomes | Both | CU | Taiwan | O | Covariate | Survival to hospital admission | Survival to hospital discharge | PH traumatic cardiac arrest with epinephrine administration | Low |
| Chen (2018) | Outcomes due to H-EMS vs G-EMS | Both | B | USA | O | Key exposure | In-hospital survival | - | H-EMS vs. GEMS transport | Low |
| Chen et al. [72] | Time vs mortality | Both | B | USA | O | Key exposure | In-hospital mortality | - | PHT | Low |
| Chen et al. [72] | Miscellaneous | Both | B | Taiwan | O | Covariate | ROSC in the ED | 30-day survival | Out of hospital traumatic cardiac arrest without PH ROSC | Low |
| Chiang et al. [70] | Effect of PH intervention on outcomes | Both | CU | Taiwan | O | Covariate | Survival to hospital admission | Survival to hospital discharge | PH traumatic cardiac arrest with epinephrine administration | Low |
| Chien (2016) | Effect of PH intervention on outcomes | Both | B | Taiwan | O | Covariate | 24-h survival | Survival to hospital discharge; cerebral function at discharge | Traumatic cardiac arrest receiving PH CPR | Low |
| Clark et al. [43] | Mortality due to rural vs urban | Both | B | USA | O | Covariate | In-hospital mortality | - | Trauma MVC | Low |
| Clements et al. [37] | Time vs mortality | Both | B | Canada | O | Key exposure | In-hospital mortality | Association between PHT and trauma team activation | All cause blunt trauma injury; EMS transport | Low |
| Crandall et al. [2] | Time vs mortality | Both | CU | USA | O | Outcome | In-hospital mortality | mean transport times | Gunshot victim > 5 miles from a trauma center | Low |
| deJongh (2012) | H-EMS: time vs mortality | Air | B | Netherlands | O | Key exposure | In-hospital mortality | - | H-EMS vs. G-EMS transport; total PH time | Low |
| DeVloo (2018) | Mortality due to primary vs secondary transfer | Both | CU | Belgium | O | Key exposure | 30-day mortality | - | Primary vs secondary transfer to tertiary center; total time to tertiary center ED; skin incision for craniotomy | Low |
| Dinh et al. [17] | Time vs mortality | Both | B | Australia | O | Key exposure | In-hospital mortality | Survival to hospital discharge without requiring transfer for rehabilitation or nursing home care | Severe TBI (AIS ≥ 3); PH time | Low |
| Fatovich et al. [66] | Mortality due to rural vs urban | Both | B | Australia | O | Key exposure | In-hospital mortality | Hospital LOS | Major trauma; rural vs urban associated PH times | Low |
| Forristal (2018) | Miscellaneous | Both | B | Canada | O | Covariate | Hypothermia (T < 35 °C) upon arrival to trauma center | Hospital LOS and survival to hospital discharge | EMS transport for severe trauma (ISS > 12) | Low |
| Foster et al. [59] | Outcomes due to H-EMS vs G-EMS | Both | B | USA | O | Covariate | Neurologic deterioration | ED disposition; in-hospital mortality; inter-facility transfer time; hospital LOS; nonroutine discharge; radiographic evidence of worsening spinal cord injury. | Spine injury with interfacility transfer; H-EMS vs G-EMS | Low |
| Franschman et al. [50] | Physiologic variables predicting outcomes in TBI | Both | B | Netherlands | O | Covariate | Neurologic deficit as determined by GOS | TBI-related mortality | TBI with transport to tertiary center; hypoxic or hypotensive events > 5 min during transport. | Low |
| Fuller et al. [79] | Time vs mortality | Both | B | UK | O | Key exposure | 30-day inpatient mortality | - | EMS transport for severe TBI (AIS-head ≥ 3); EMS PHT intervals | Low |
| Fuller et al. [51] | Physiologic variables predicting outcomes in TBI | Both | B | UK | O | Key exposure | In-hospital mortality | Vital sign deterioration | TBI with transport to tertiary center; PHT intervals | Low |
| Funder et al. [40] | Time vs mortality | Both | CU | Denmark | O | Key exposure | 30-day mortality | - | Penetrating trauma by EMS to trauma center; PHT | Low |
| Garcia (2017) | Time vs mortality | G-EMS | CU | Canada | O | Key exposure | In-hospital mortality | - | Trauma with EMS transport to trauma center; PHT in intervals | Low |
| Gauss et al. [21] | Time vs mortality | Both | B | France | O | Key exposure | In-hospital mortality | - | Physician-staffed EMS to trauma center; PHT in intervals | Low |
| Gomes (2010) | Effect of PH intervention on outcomes | Both | B | Portugal | O | Covariate | In-hospital mortality | - | Severe trauma requiring procedure; procedure done in PH; first hospital; arrival to trauma center | Low |
| Haltmeier et al. [52] | Effect of PH intervention on outcomes | Both | B | USA | O | Outcome | In-hospital mortality | Ventilator days; length of ICU stay; on-scene; PH time | Isolated severe blunt head injury (PH GCS ≤ 8) with or without PH intubation | Low |
| Hesselfeldt et al. [61] | H-EMS: mortality from physician vs paramedic | Air | B | Denmark | O | Outcome | Time from dispatch first ground EMS to arrival in the TC trauma bay | Proportion of severely injured patients secondarily transferred to the trauma center; 30-day mortality; on-scene triage. | Severe trauma patient transported by MD staffed H-EMS; PH fluid administration | Low |
| Hussmann et al. [63] | Effect of PH intervention on outcomes | Both | B | Germany | O | Covariate | In-hospital mortality | Sepsis; organ failure; multiple organ failure | Trauma with bleeding requiring transfusion > 1 unit pRBCs in hospital; PH fluid administration | Moderate |
| Hussmann et al. [64] | Effect of PH intervention on outcomes | Both | B | Germany | O | Covariate | In-hospital mortality | Hospital LOS; ICU LOS; ICU intubation; sepsis; organ failure; multi-organ failure | Level of PH fluid resuscitation of severe TBI patients | Low |
| Ingalls et al. [80] | H-EMS: time vs mortality | Air | C | Multiple | O | Key exposure | 30-day mortality | Mortality en-route | Rapid evacuation by the Critical Care Air Transport (CCATT): time from wounding until time of arrival at the definitive care facility | Low |
| Jung et al. [38] | H-EMS: mortality from physician vs paramedic | Air | CU | South Korea | O | Covariate | Survival | TRISS | Group P patients transported by physician-staffed HEMS and group NP patients were transported by nonphysician-staffed HEMS | Low |
| Karrison (2018) | G-EMS: time/distance vs mortality | G-EMS | CU | USA | O | Key exposure | ED/hospital mortality | None | Driving distance (shortest driving distance from the geocoded location of the scene of injury to the trauma center) transport time | Moderate |
| Kidher et al. [81] | H-EMS: time vs mortality | Air | CU | England | O | Key exposure | Mortality |  | Time-related variables, stay on scene time, arrival on scene time, total scene time | Moderate |
| Kim et al. [25] | Effect of PH intervention on outcomes | Air | CR | USA | I | Covariate | Mortality (overall and 24-h mortality) | Hospital stay; ICU LOS; ARDS, ARF | PH plasma administration | Moderate |
| Kim et al. [82] | G-EMS: time/distance vs mortality | G-EMS | Not specified | South Korea | O | Key exposure | In-hospital mortality |  | Scene time, PHT | Low |
| Klein (2019) | Time vs mortality | Both | B | Multiple | O | Key exposure |  | Early SURG; ICU LOS; days intubated; organ failure; multiple organ failure; sepsis RISC prognosis; TRISS prognosis; in-hospital mortality; death within the first hour; death within the first 24 h; days of hospitalization | PH treatment time by intervals | Moderate |
| Kotwal et al. [32] | H-EMS: time vs mortality | Air | C | Afghanistan | O | Key exposure | Overall mortality, killed in action mortality, died of wound mortality | Amputation; cardiac arrest; coagulopathy; shock | Helicopter time < 60 min vs > 60 min | Moderate |
| Kotwal et al. [73] | Time vs mortality | Both | C | Multiple | O | Key exposure | Mortality |  | PH transport time, injury severity, blood transfusion | Moderate |
| Kulla et al. [60] | Miscellaneous | Both | B | Germany | O | Outcome | Trauma resuscitation time prolongation |  | Invasive emergency procedures | Low |
| Lansom et al. [53] | Effect of PH intervention on outcomes | Both | B | Australia | O | Outcome | Survival | Reduction in time from ED arrival to CT imaging | PH intubation compared with ED intubation | Low |
| Leis (2013) | Effect of PH intervention on outcomes | G-EMS | CU | Spain | O | Key exposure | Survival to discharge |  | Response time | Low |
| Lovely et al. [44] | G-EMS: time/distance vs mortality | G-EMS | CR | USA | O | Key exposure | In-hospital mortality |  | PH scene time, PH transport time, Injury Severity Score (ISS) | Low |
| Lyon et al. [26] | Effect of PH intervention on outcomes | Air | B | England | I | Covariate | Mortality | ICU LOS | PRBC Transfusion | Low |
| Maddry et al. [46] | H-EMS: time vs mortality | Both | C | Not specified | O | Key exposure | Mortality up to 30 days | Morbidity up to 30 days, ICU and hospital stay | Time from the initial request for medical evacuation to arrival at a medical treatment facility | Moderate |
| Majidi et al. [54] | Physiologic variables predicting outcomes in TBI | Both | CU | USA | O | Covariate |  | Total hospital stay; in-hospital mortality; intensive care unit (ICU) days; ventilator days; discharge destinations | PH Neurologic Deterioration PHND | Moderate |
| Malekpour et al. [65] | Mortality due to primary vs secondary transfer | Both | CR | USA | O | Covariate | In-hospital mortality, ICU LOS, hospital LOS, complications | Pneumonia; pulmonary embolus; deep venous thrombosis; major arrhythmia, urinary tract infection, wound infection, acute renal failure | DA-direct admission  IHT-Interhospital transfer | Moderate |
| McCoy (2013) | G-EMS: time/distance vs mortality | G-EMS | CU | USA | O | Key exposure | In-hospital mortality |  | EMS on-scene and transport time intervals | Moderate |
| Meizoso et al. [71] | Effect of PH intervention on outcomes | Both | CU | USA | O | Outcome | Mortality on arrival (or DOA) |  | Intubation, needle decompression, tourniquet use, cricothyroidotomy, or advanced cardiac life support | Low |
| Middleton (2012) | Miscellaneous | Both | B | Australia | O | Key exposure | Short-term neurological recovery (as determined by patient’s ASIA impairment scale grade on discharge from SCIU) | Deep vein thrombosis; pulmonary embolism; pressure ulcers | Time to definitive care center SCIU | Low |
| Möller et al. [22] | G-EMS: time/distance vs mortality | G-EMS | CU | South Africa | O | Key exposure | Mortality |  | Method of transport, hospital arrival time or PH transport time intervals | Low |
| Moore et al. [27] | Effect of PH intervention on outcomes | G-EMS | CU | USA | I | Outcome | Mortality | MOF at 28 days trauma-induced coagulopathy  Shock  Acute lung injury  Exploratory outcomes: time from injury to need for first red blood cell transfusion  Thromboelastography indices  Number of ventilation free days  Number of intensive-care-free days  Development of MOF | Plasma administered in PH setting within 30 min of injury | High |
| Murad et al. [34] | G-EMS: time/distance vs mortality | G-EMS | B | Iraq | O | Key exposure | Mortality | Physiologic Severity Score | Assess 2 tier PH system (first responder and paramedic) vs no EMS in patients with long PHTs | Low |
| Murad et al. [35] | G-EMS: time/distance vs mortality | G-EMS | B | Iraq | O | Key exposure | Mortality |  | PH period intervals | Low |
| Neeki, et al. [28] | Effect of PH intervention on outcomes | Both | B | USA | I | Outcome | Mortality 24 h, 48 h, and 28 days | Total blood products transfused  Hospital and ICU LOS, SBP prior to TXA administration, GCS prior to the first TXA dose in the field  Adverse events | Prehospital TXA administration vs no TXA administration in patients with signs of h. shock | High |
| Newberry (2019) | Miscellaneous | G-EMS | CR | India | O | Covariate | Mortality at 2, 7, and 30 days | Oxygen delivery; Intravenous fluids; functional status | Transport by EMS if burn injury | Low |
| Newgard et al. [55] | Outcomes due to H-EMS vs G-EMS | Both | B | Multiple | O | Key exposure | 28-day mortality in shock, 6-month neurologic function in TBI |  | Total out-of-hospital time (time of initial 9-1-1 call to time of EMS arrival at the receiving hospital ED) | Moderate |
| Newgard (2010) | Outcomes due to H-EMS vs G-EMS | Both | B | Multiple | O | Key exposure | Mortality |  | EMS time intervals | Moderate |
| Pakkanen et al. [56] | G-EMS: mortality from physician vs paramedic | Both | B | Finland | O | Covariate | Mortality, neurological outcome of TBI patients |  | EMS physician-staffed, EMS paramedic-staffed | Low |
| Paravar (2014) | G-EMS: time/distance vs mortality | G-EMS | B | Iran | O | Key exposure | Mortality (in-hospital) |  | PHT advanced trauma life support interventions | Low |
| Prabhakaran et al. [57] | Mortality due to primary vs secondary transfer | Both | CU | USA | O | Outcome | Mortality in TBI | Time to arrival at a level I trauma center; time to initiation of multimodality neurophysiological monitoring; goal-directed therapy protocol | Scene to hospital vs transfer to hospital | Low |
| Pusateri et al. [29] | Effect of PH intervention on outcomes | Both | B | USA | I | Covariate | 28-day mortality | 24-h mortality; volumes of in-hospital blood components administered; ventilator-free days | PH transport times  COMBAT Study pt. received plasma vs standard care  PAMPer Study pt. received plasma vs standard care | Moderate |
| Raatiniemi (2015) | Mortality due to rural vs urban | Air | B | Finland | O | Covariate | 30-day mortality rate | Length of intensive care unit stay | Rural vs urban HEMS | Low |
| Rappold et al. [41] | Miscellaneous | G-EMS | CU | USA | O | Covariate | Mortality in hospital |  | ALS-transported trauma victims relative to BLS-transported trauma victims and among police-transported trauma victims | Low |
| Reitz et al. [30] | Effect of PH intervention on outcomes | Both | B | USA | I | Outcome | 28-day mortality | 24-h mortality; PH transport time; presenting indices of shock and coagulopathy units of in-hospital blood components administered | COMBAT study pt. received plasma vs standard care  PAMPer Study pt. received plasma vs standard care | Moderate |
| Ruelas (2018) | Time vs mortality | Both | B | USA | O | Key exposure | PH and ED mortality |  | PHT and procedures on penetrating trauma | Low |
| Ryb (2013) | Outcomes due to H-EMS vs G-EMS | Both | B | USA | O | Covariate | Mortality |  | HEMS VS GEMS | Low |
| Seamon et al. [42] | Time vs mortality |  | CU | USA | O | Key exposure | Mortality |  | PHT prolonged by ALS vs BLS | Low |
| Shackelford et al. [33] | Effect of PH intervention on outcomes | Air | C | Afghanistan | O | Key exposure | Mortality at 24 h and 30 days | Prevalence of shock | Initiation of PH transfusion RBC, plasma, or both | Moderate |
| Spaite et al. [58] | Physiologic variables predicting outcomes in TBI | Both | CU | USA | O | Key exposure | Mortality in-hospital |  | Hypotension depth-duration out of hospital | Moderate |
| Talving (2009) | Outcomes due to H-EMS vs G-EMS | Both | CU | USA | O | Covariate | Mortality | LOS; discharge time; ICU admission | HEMS vs. ground emergency medical service (GEMS) > 30 min | Low |
| Tansley (2019) | G-EMS: time/distance vs mortality | G-EMS | B | Canada | O | Key exposure | Mortality |  | PH transfer time to trauma center | Low |
| Taylor (2018) | Outcomes due to H-EMS vs G-EMS | Both | B | USA | O | Covariate | Mortality |  | HEMS vs. ground emergency medical service (GEMS) | Low |
| Tien (2011) | G-EMS: time/distance vs mortality | G-EMS | CU | Canada | O | Key exposure | Hospital survival |  | PHT  Time-to-surgery | Low |
| Weichenthal (2015) | Effect of PH intervention on outcomes | Both | B | USA | O | Covariate | Survival to hospital discharge |  | Needle thoracostomy VS No Needle Thoracostomy | Low |
| Yeguiayan et al. [39] | G-EMS: mortality from physician vs paramedic | G-EMS | CU | France | O | Covariate | 30-day mortality | 72-h mortality | Physician EMS vs non-Physician EMS | Low |
| Zalstein (2010) | Miscellaneous | Both | B | Australia | O | Covariate | Mortality | Adverse events | Patient inter-hospital transfer | Low |
| Zhu (2019) | Miscellaneous | Both | B | USA | O | Covariate | Survival, LOS, ICU days, ventilator days |  | Pt that required mass transfusion protocol | Low |
| Zhu (2018) | Outcomes due to H-EMS vs G-EMS | Both | CR | USA | O | Covariate | Survival to discharge from hospital |  | HEMS v GEMS | Low |
